# Molecular insight into cotton leaf curl geminivirus disease resistance in cultivated cotton (*Gossypium hirsutum*)

**DOI:** 10.1111/pbi.13236

**Published:** 2019-09-30

**Authors:** Syed Shan‐e‐Ali Zaidi, Rubab Zahra Naqvi, Muhammad Asif, Susan Strickler, Sara Shakir, Muhammad Shafiq, Abdul Manan Khan, Imran Amin, Bharat Mishra, M. Shahid Mukhtar, Brian E. Scheffler, Jodi A. Scheffler, Lukas A. Mueller, Shahid Mansoor

**Affiliations:** ^1^ National Institute for Biotechnology and Genetic Engineering Faisalabad Pakistan; ^2^ Boyce Thompson Institute Ithaca NY USA; ^3^ Plant Genetics Lab TERRA Teaching and Research Center Gembloux Agro-Bio Tech University of Liège Gembloux Belgium; ^4^ Department of Biology University of Alabama at Birmingham Birmingham AL USA; ^5^ Genomics and Bioinformatics Research Unit United States Department of Agriculture‐Agricultural Research Service (USDA‐ARS) Stoneville MS USA; ^6^ Crop Genetics Research Unit United States Department of Agriculture‐Agricultural Research Service (USDA‐ARS) Stoneville MS USA; ^7^ Present address: Department of Biotechnology University of Okara Okara Pakistan

**Keywords:** *Gossypium hirsutum*, leaf curl disease, plant virus resistance, transcriptome, WGCNA

## Abstract

Cultivated cotton (*Gossypium hirsutum*) is the most important fibre crop in the world. Cotton leaf curl disease (CLCuD) is the major limiting factor and a threat to textile industry in India and Pakistan. All the local cotton cultivars exhibit moderate to no resistance against CLCuD. In this study, we evaluated an exotic cotton accession Mac7 as a resistance source to CLCuD by challenging it with viruliferous whiteflies and performing qPCR to evaluate the presence/absence and relative titre of CLCuD‐associated geminiviruses/betasatellites. The results indicated that replication of pathogenicity determinant betasatellite is significantly attenuated in Mac7 and probably responsible for resistance phenotype. Afterwards, to decipher the genetic basis of CLCuD resistance in Mac7, we performed RNA sequencing on CLCuD‐infested Mac7 and validated RNA‐Seq data with qPCR on 24 independent genes. We performed co‐expression network and pathway analysis for regulation of geminivirus/betasatellite‐interacting genes. We identified nine novel modules with 52 hubs of highly connected genes in network topology within the co‐expression network. Analysis of these hubs indicated the differential regulation of auxin stimulus and cellular localization pathways in response to CLCuD. We also analysed the differential regulation of geminivirus/betasatellite‐interacting genes in Mac7. We further performed the functional validation of selected candidate genes via virus‐induced gene silencing (VIGS). Finally, we evaluated the genomic context of resistance responsive genes and found that these genes are not specific to A or D sub‐genomes of *G. hirsutum*. These results have important implications in understanding CLCuD resistance mechanism and developing a durable resistance in cultivated cotton.

## Introduction

Cotton (*Gossypium* spp.) is the most important fibre‐producing plant in the world; it not only provides fibre for the textile industry but also contributes to a significant portion of animal feed and edible oil. In terms of production, China, the United States, India and Pakistan are the world's major cotton producing countries. In the Indian subcontinent, cotton is facing a real threat due to cotton leaf curl disease (CLCuD), one of the most important diseases and a limiting factor of cotton production in India and Pakistan (Sattar *et al*., 2013). CLCuD is caused by whitefly (*Bemisia tabaci*)‐transmitted single‐stranded (ss) DNA viruses of the genus *Begomovirus* (family *Geminiviridae*). Based on their genomic components, begomoviruses are classified in two groups, monopartite and bipartite. The genomes of monopartite and DNA‐A components of bipartite begomoviruses encode the coat protein (CP), V2/AV2 protein in the virion‐sense orientation and the replication‐associated protein (Rep; a rolling circle replication initiator protein), the replication enhancer protein (REn), the transcriptional activator protein (TrAP) and the C4 protein in the complementary‐sense orientation (Fondong, 2013). DNA‐B components encode the nuclear shuttle protein (NSP) and movement protein (MP) in the virion‐ and complementary‐sense, respectively. The reading frames in the virion‐ and complementary‐sense of begomovirus genomes/genomic components are separated by a noncoding (intergenic) region which contains cis‐acting regulatory elements for gene expression and a predicted hairpin structure. The hairpin structure contains the conserved (among most geminiviruses) nonanucleotide sequence TAATATTAC as part of the loop and small repeated sequences, known as ‘iterons’, which are sequence‐specific binding sites for the Rep protein. Together, the iterons and hairpin form the origin of replication (*ori*) for virion‐sense viral DNA replication. The majority of monopartite begomoviruses are associated with additional small ssDNA molecules known as betasatellites and alphasatellites (Briddon and Stanley, 2006). Betasatellites (previously known as DNA‐β) are half the size of begomovirus components (∼1350 nt) and encode a single gene in the complementary‐sense that codes for an ∼118 amino acids protein known as βC1. Betasatellites may increase the accumulation of their helper begomoviruses, as well as enhance symptoms in some host plants (Briddon *et al*., 2001; Zhou, 2013). This is likely due to βC1 having suppressor of RNA interference activity (Cui *et al*., 2005; Yang *et al*., 2011). The alphasatellites [previously known as DNA‐1; (Briddon *et al*., 2004)] are not strict satellites, since they are capable of autonomous replication in permissive host plants. They are dependent on their helper begomoviruses for movement within plants and insect transmission between plants (Mansoor *et al*., 1999; Saunders and Stanley, 1999). Although widespread in the Old World (OW), alphasatellites have also been identified in the New World (NW) in association with bipartite begomoviruses, in the absence of betasatellites (Paprotka *et al*., 2010; Romay *et al*., 2010).

Since its first identification in the 1960s, CLCuD in the southern Asia has gone through four so called four phases—pre‐epidemic, epidemic, resistance breaking and postresistance breaking. Each of these phases for which information is available was/is associated with distinct viruses but all are characterized by the presence of a single betasatellite species—*Cotton leaf curl Multan betasatellite* (CLCuMuB) (Zubair *et al*., 2017a). While cotton yield in Pakistan topped a record high during 1991–1992 cropping cycle, it suffered tremendously owing to CLCuD epidemic during 1992–1993 (Mahmood, 1999). This epidemic phase was associated with the presence of multiple begomovirus species, the most common of which were *Cotton leaf curl Multan virus* and *Cotton leaf curl Kokhran virus* (CLCuKoV) (Mansoor *et al*., 2003). Towards the end of the epidemic phase, CLCuD almost entirely disappeared from cotton as resistant cotton varieties, developed by conventional breeding, came into widespread cultivation by farmers. The beginning of the resistance breaking phase can be pinpointed precisely in the early 2000s with the appearance of a very unusual begomovirus strain, CLCuKoV‐Burewala (CLCuKoV‐Bu; previously known as *Cotton leaf curl Burewala virus*), a recombinant of CLCuKoV and CLCuMuV. The resistance breaking strain was also associated with a recombinant version of CLCuMuB (Amrao *et al*., 2010). The postresistance breaking phase has witnessed a slow shift from CLCuKoV‐Bu to a situation more akin to that of the epidemic phase, with at least some of the earlier virus species/strains reappearing in cotton (Zubair *et al*., 2017b). Although other begomoviruses, and even a mastrevirus (leafhopper‐transmitted geminivirus), were sporadically reported in cotton during all the phases for which information is available (Hameed *et al*., 2014a,b; Zaidi *et al*., 2015, 2016), the disease was always associated with CLCuMuB which is essentially required for symptom development (Zubair *et al*., 2017a). A large collection of available germplasm has been screened in search of resistance against CLCuD and several lines identified as resistant to CLCuD complex have since become susceptible to CLCuD (Ahmad *et al*., 2010). In this scenario, where local cotton varieties in Pakistan were highly susceptible to CLCuD, the search to find resistant cotton was expanded to include accessions from the United States Department of Agriculture (USDA) cotton germplasm collection and USDA Agricultural Research Service (ARS) breeding programs.

Mac7 (GVS9) came from a breeding program to select for budworm (*Heliothis virescens*) resistance. It was developed by first making a cross between a parent from USDA that had a high level of gossypol and reportedly insect tolerance traits introgressed from *Gossypium raimondii* and XG‐15 parent. XG‐15 is a cotton line with a high level of gossypol and, in replicated tests, was reported to inhibit *H. virescens* larval growth. XG‐15 originated from a cross between a wild *G. hirsutum* strain from Socorro Island and Deltapine 15 (PI 52870). A selected F2 progeny from that cross was then crossed to M‐11 (a doubled haploid of Empire PI 529179) and progeny plants selected for resistance to *H. virescens* resistance, based on replicated caged tests. Resistant progeny from the cross were bulked to create the original Mac7, which segregated for a number of phenotypic characteristics. Three rounds of single plant selection were made to the original Mac7 to improve its phenotypic characteristics and a selection released as Mac7 (GVS9). This selection had a high level of gossypol, with 90% in the plus form, which has been associated with enhanced insect and disease tolerance. Mac7 (GVS9) was not previously evaluated for disease resistance.

The availability of cotton genome *G. raimondii* (Paterson *et al*., 2012; Wang *et al*., 2012), *G. arboreum* (Li *et al*., 2014) and upland *G. hirsutum* (Li *et al*., 2015; Zhang *et al*., 2015) sequences has enabled the use of RNA‐Seq for cotton transcriptomic studies. Previous studies have shown the efficacy of transcriptomic and proteomic data sets for understanding the molecular mechanisms of begomovirus and whitefly infestation on tomato and cultivated cotton *G. hirsutum* (Li *et al*., 2016; Zhao *et al*., 2019), cassava mosaic begomovirus infection on cassava (Allie *et al*., 2014), tomato yellow leaf curl virus (TYLCV) infection on tomato (Chen *et al*., 2013; Hasegawa *et al*., 2018; Huang *et al*., 2016), geminivirus infection on pepper (Gongora‐Castillo *et al*., 2012) and other pathways related to begomovirus (Brustolini *et al*., 2015) and cotton development (Hu *et al*., 2018).

In this study, we screened *G. hirsutum* accession Mac7 for CLCuD resistance and performed qPCR for identification and quantification of the CLCuD complex in Mac7. We then utilized RNA‐Seq for identification of CLCuD responsive genes in Mac7 and validated our data with qPCR. After ensuring the high quality, validity and reproducibility of the RNA‐Seq data, we performed several downstream analyses such as weighted co‐expression gene network analysis, gene ontology, KEGG pathway analysis, regulation of R‐genes and geminivirus/betasatellite‐interacting genes. We further performed functional validation via virus‐induced gene silencing (VIGS) on selected candidate genes. Our results indicate the molecular mechanism of CLCuD resistance with reference to genomic context of resistance genes in cotton.

## Results and discussion

### Mac7 is resistant to CLCuD and associated with the absence of betasatellite

**Figure 1:**
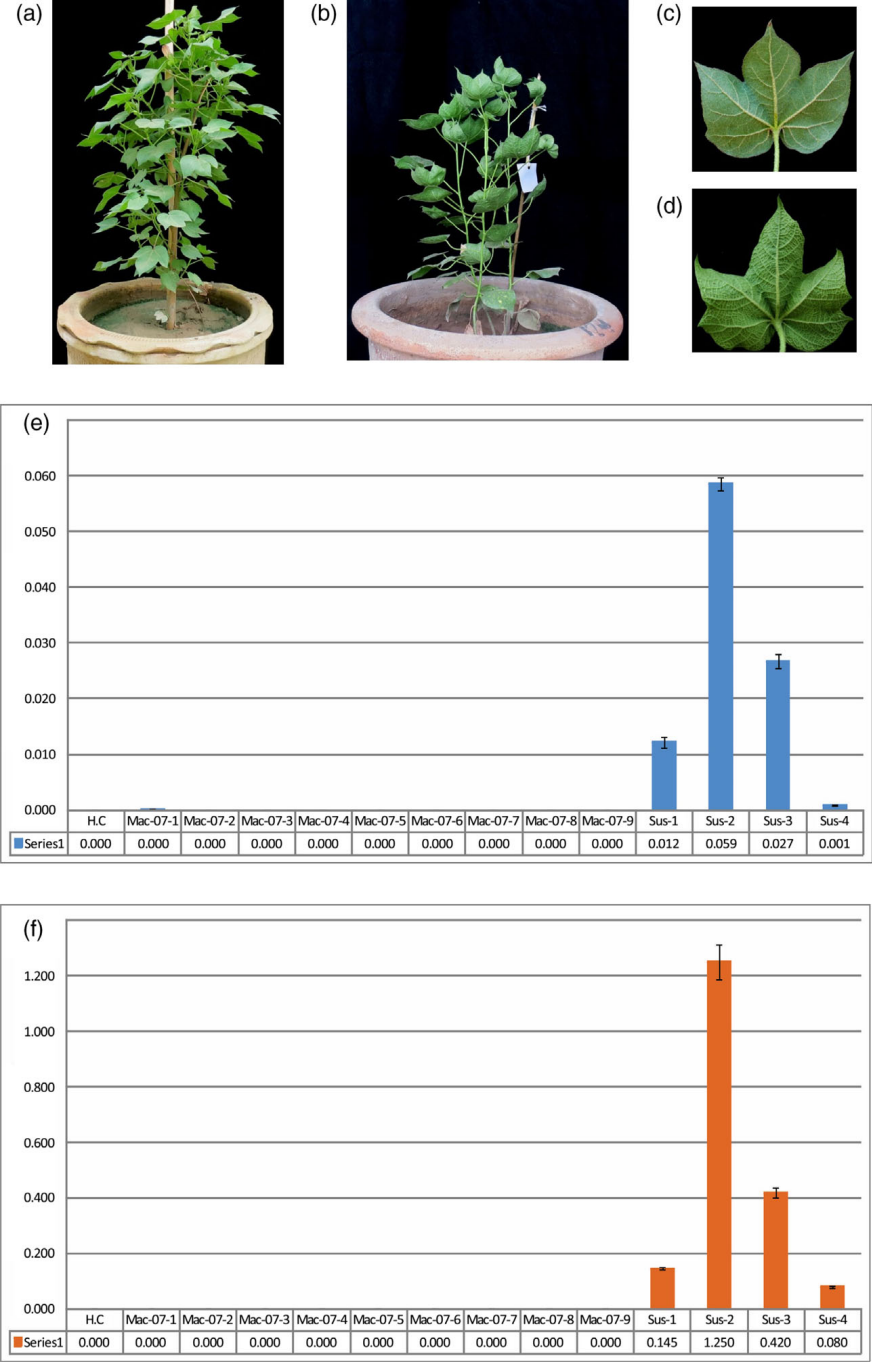
Cotton leaf curl disease (CLCuD) symptoms and quantification on resistance and susceptible cotton genotypes. CLCuD‐resistant *Gossypium hirsutum* accession Mac7 plant (a) and CLCuD‐susceptible *G. hirsutum* accession karishma plant (b) with close view of back side of single leaves of Mac7 (c) and karishma (d). No symptoms of CLCuD observed on Mac7 where karishma maintained in the same glasshouse under the infestation of viruliferous whiteflies developed severe CLCuD symptoms. Characteristic CLCuD symptoms including leaf curling, leaf vein thickening and stunted growth can be clearly observed on susceptible cotton. Identification and quantification of CLCuD‐associated begomoviruses (e) and betasatellite (f) in susceptible and resistant cotton. qPCR with begomovirus and betasatellite primers is represented with blue and orange colours, respectively. HC represents healthy control. Nine samples of Mac7 (Mac‐07‐01–Mac‐07‐09) and four samples of susceptible genotype (Sus‐1–Sus‐4) were used in qPCR experiment. *X*‐axis indicates the concentration of respective component's concentration in nanograms per microgram of plant genomic DNA.

Around 5000 upland cotton accessions from the United States Department of Agriculture (USDA) cotton germplasm collection and USDA Agricultural Research Service (ARS) breeding programs were screened for CLCuD resistance in Pakistan (ICARDA, 2015, 18–19 August). Screening performed at three different locations (Faisalabad, Vehari and Multan) indicated that an accession Mac7 (GVS9, ARS release number P.0063.14) remained resistant to CLCuD, where the local genotypes (such as CIM‐446, CIM‐496, AA‐703, AA‐802, MNH‐886 and CIM‐599) and other exotic germplasm developed characteristic CLCuD symptoms (Shah *et al*., 2017). We evaluated Mac7 for CLCuD resistance under glasshouse conditions, in comparison with CLCuD susceptible *G. hirsutum* cultivar Karishma maintained in whitefly‐infested conditions and monitored regularly for CLCuD symptoms. All 40 Mac7 plants remained asymptomatic for CLCuD throughout the whole lifecycle. Karishma plants, on the other hand, started showing characteristic CLCuD symptoms such as curling of leaf margins, leaf vein thickening, cup‐shaped leaves, leaf‐like enations under leaves and stunned growth, 14–21 days postgermination (Figure 1a–d). These results, in combination with the field trials, confirmed that Mac7 is resistant to CLCuD.

For the identification and quantification of CLCuD begomoviruses and associated betasatellites, we performed qPCR on resistant and susceptible genotypes of *G. hirsutum*. qPCR is recently optimized as a standard method for quantification of begomoviruses and associated satellites causing CLCuD in cotton (Shafiq *et al*., 2017). qPCR with begomovirus‐specific primers indicated that while high levels of begomovirus are maintained in susceptible cotton, comparatively very low level of the virus was detected in Mac7 plants (Figure 1e). Interestingly, qPCR with betasatellite specific primers indicated that where CLCuMB was detected in susceptible cotton, it was absent in Mac7 (Figure 1f). Also, while comparing begomovirus and betasatellite levels in susceptible and resistant cotton using qPCR, the most abundant component in susceptible cotton was betasatellite (Figure S5). In the case of CLCuD, CLCuMuB is the pathogenicity determinant (Mansoor *et al*., 2003); it is the most important component for CLCuD and is often necessary for disease symptom induction (Briddon *et al*., 2014). The absence of betasatellite in Mac7 can therefore be linked to the absence of CLCuD symptoms and low levels of begomoviruses in Mac7.

### Identification and validation of genes associated with CLCuD resistance in Mac7

**Figure 2:**
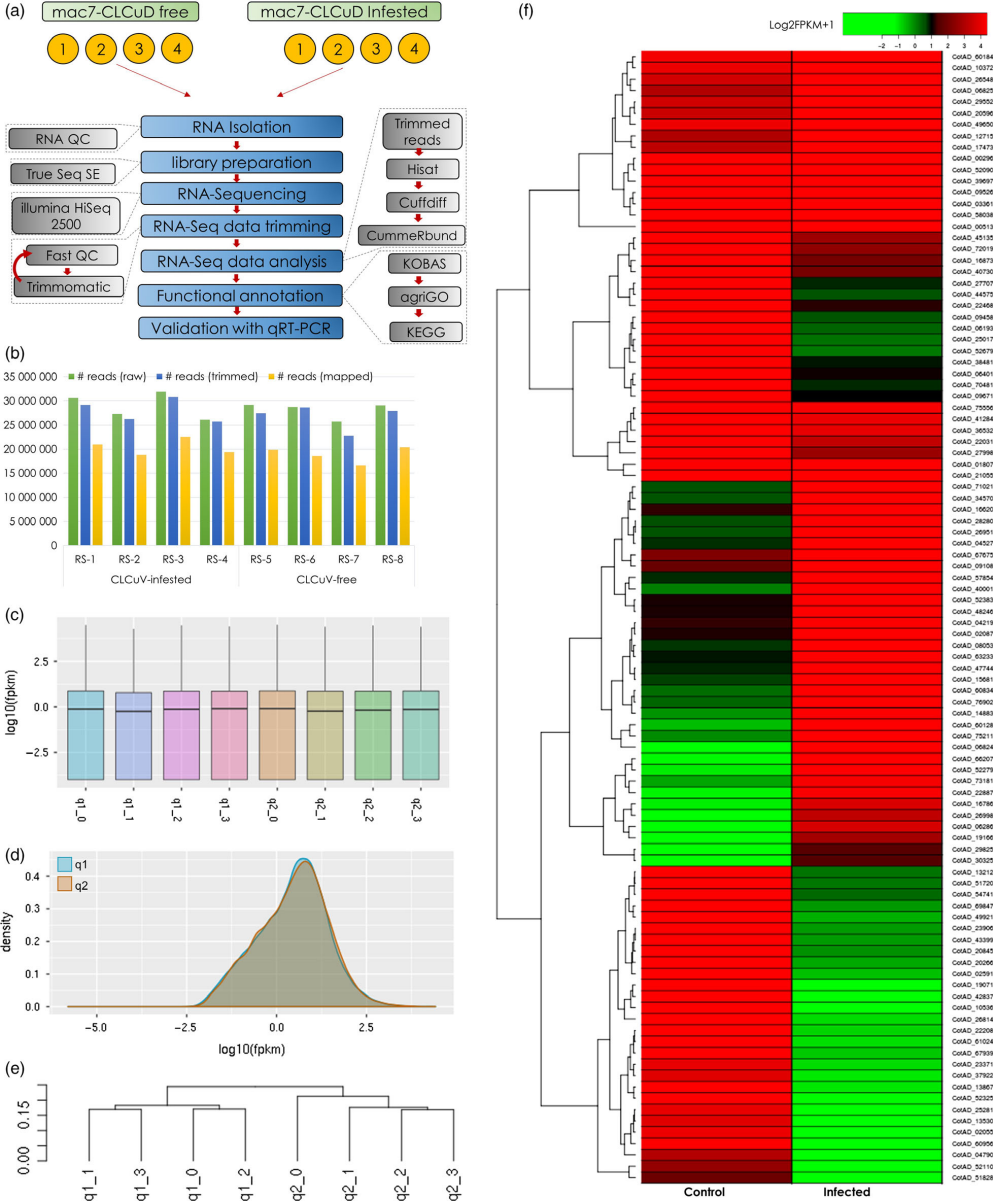
RNA‐Seq pipeline and transcriptomic data analysis. (a) Schematic representation of RNA‐Seq workflow for Mac7 with and without the infestation of cotton leaf curl disease (CLCuD). Four biological replicates were used in each treatment. (b) RNA‐Seq reads mapped/unmapped and mapping percentage for each sample. Panel (c) and (d) indicate the distribution of log10 of FPKM (fragments per kilobase of transcript per million mapped reads) in each sample where q1 represents Mac7 CLCuD free and q2 represents Mac7 under CLCuD infestation. Panel (e) represents the phylogenetic tree based on log10FPKMs of each sample. Clear distribution of q1 and q2 in separate clades indicate that biological replicates for each treatment are true representatives of each condition and assure the reproducibility of experiment. Panel (f) shows the heatmap of differentially expressed genes (DEGs) in RNA‐Seq data of Mac7 under CLCuD infestation (Column 2: infected) as compared to CLCuD‐free Mac7 (Column 1: Control). Log2FPKM + 1 value was used to construct heatmap as indicated on the colour key; heatmap2 package in R was used to construct heatmap.

To understand the mechanism of CLCuD resistance in Mac7 and identification of differentially expressed genes (DEGs), a transcriptome‐wide analysis was performed on CLCuD‐infested and CLCuD‐free Mac7. While analysing the sequencing results, quality check measures were performed on each step to ensure reproducibility of the experiment (Figure 2a). We compared individual sequencing runs from each condition and calculated the transcript abundances of each run in terms of FPKMs (fragments per kilobase of transcript per million mapped reads) (Figures 2c and S2) that provided standard gene density and dispersion (Figures 2d and S3). We then constructed a phylogenetic dendrogram based on log10FPKMs, which clearly separated the reads from CLCuD‐infested and CLCuD‐free samples in separate clusters (Figure 2e) highlighting the confidence on biological replicates.

**Figure 3:**
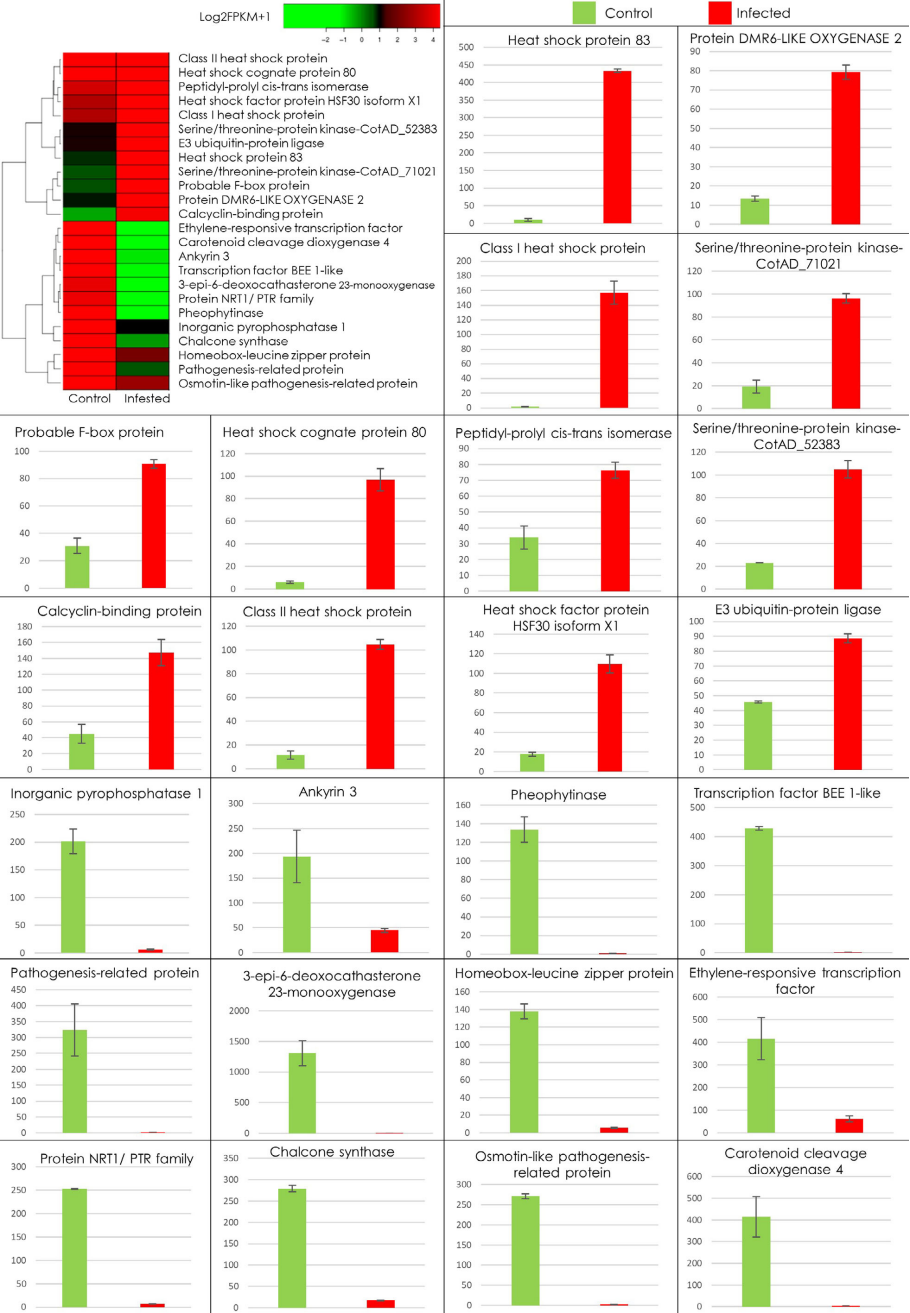
Significant differentially expressed genes in Mac7 transcriptome and their validation with qPCR. Each graph in the figure indicates the level of the respective gene quantified using qPCR. In each graph, the green bar indicates the RNA level in CLCuD‐free Mac7, while red bar indicates the RNA level in CLCuD‐infected Mac7. Error bar indicates the variation among three replicates for each gene. The heatmap on the top left of figure provides the level of respective genes in RNA‐Seq data and quantified on base of Log2FPKM + 1.

On average, 30 million raw reads were obtained from each replicate (Figure 2b), which were further trimmed to ~27 million, retaining only high quality reads with an average per base Phred score of 37 (Figures 2b and S1). Among these, ~72% reads per sample mapped to the *G. hirsutum* genome (Table S1). Comparing the transcriptome of CLCuD infested Mac7 (samples RS1‐RS4) with the CLCuD free Mac7 (RS5‐RS8) yielded significant differentially expressed genes (Figure S4 and Table S3). We also generated transcriptomic data set on CLCuD‐susceptible line karishma and compared the data set with Mac7 using the same pipeline explained in the methods section. The data analysis yielded a huge number (>2400) DEGs (Table S8). The difference between the origin and physiology of karishma (a Pakistani local cotton breed) and Mac7 (USDA germplasm) is a probable explanation of these huge DEG data sets. Therefore, we have focused on Mac7 non‐virus‐infected versus Mac7 virus‐infected data sets. For validation of identified DEGs in Mac7 RNA‐Seq, qPCR was performed on 24 DEGs selected on the basis of log2 fold change, *P*‐values and q‐values (Table S10). qPCR indicated that the genes, significantly up‐regulated in RNA‐Seq data, were also up‐regulated in qPCR, and vice versa (Figure 3). These results ensured the validity of the RNA‐Seq data, which were used in downstream co‐expression and annotation analysis. Furthermore, to ensure the unbiased identification of DEGs, eight housekeeping genes in *G. hirsutum* were evaluated for differential expression (Artico *et al*., 2010). None of these eight reference genes were differentially expressed (Table S2), further validating the authenticity of transcriptomic data.

### WGCNA identified nine novel modules associated with defence response

**Figure 4:**
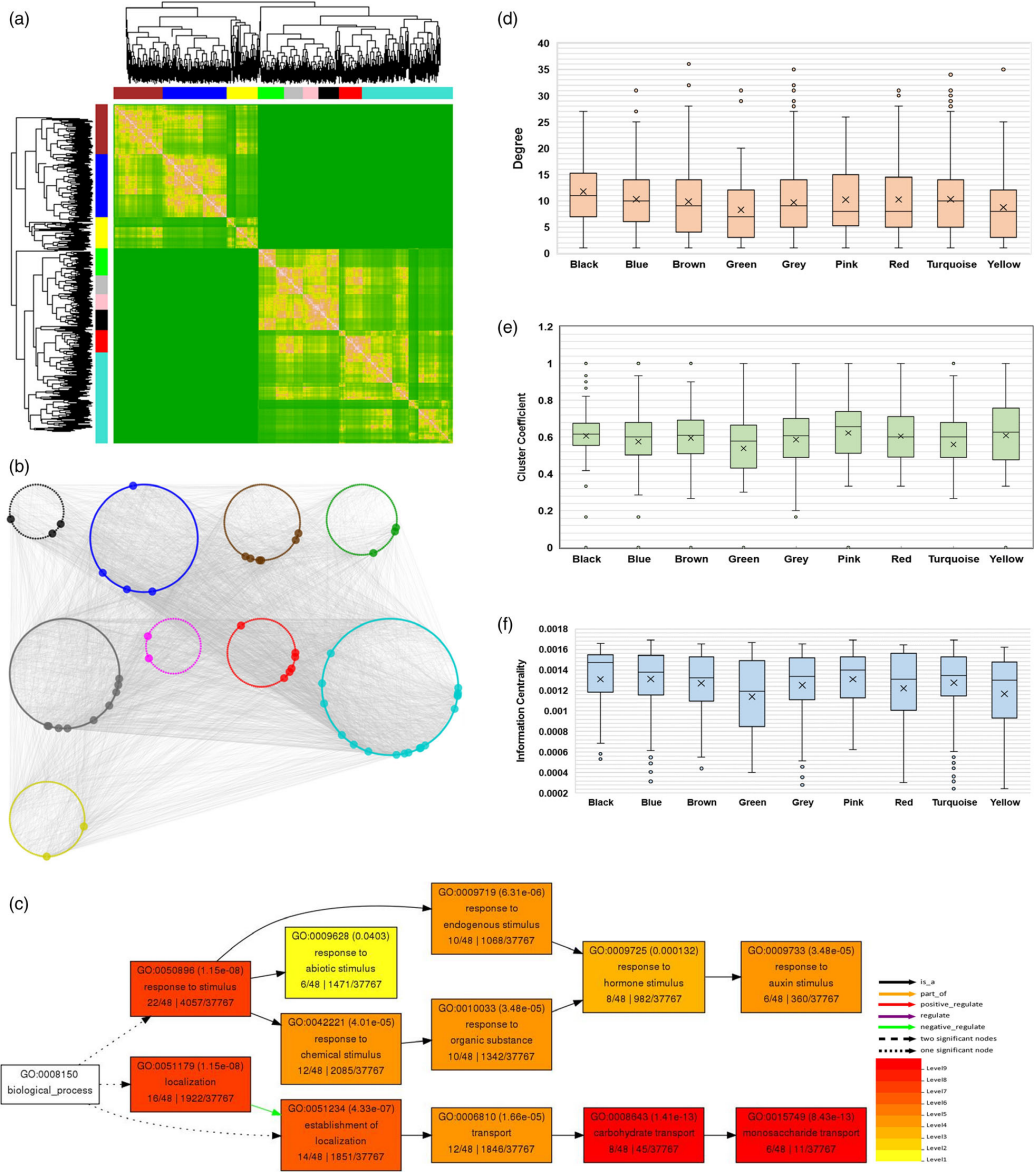
Weighted gene co‐expression network construction and co‐expressed network analysis for CLCuD‐responsive cotton genes in resistant genotype. (a) Resistant cotton genotype Mac7, under CLCuD infestation and CLCuD‐free conditions, was analysed by WGCNA using 1676 DEGs. Genes were clustered as per expression arrangements characterized by the dendrogram and topological overlap mapping metric (TOM) heatmap. Each line of the dendrogram corresponds to a gene. Clusters of similarly regulated genes are indicated as modules by a corresponding colour (black, blue, brown, green, grey, pink, red, turquoise and yellow) with a threshold minimum module size of 70 genes. The intensity of pink colouring in heatmap specifies high strength and green as no strength of correlation between pairs of genes on a direct scale and features hierarchical clustering dendrograms possessing a range of weighted correlations. (b) Weighted network illustrates correlations (edges) among the nodes (genes) with a weighted correlation threshold of ≥0.85. The network is composed of 7770 connections and 1564 genes organized in nine different modules. The node colour corresponds to modules identified via WGCNA. Nodes with high connectivity (≥25 connections, hubs) among different modules are indicated with increased node size. (c) Gene ontology analysis of gene IDs associated with highly correlated 52 hubs among 9 modules identified in WGCNA. GO analysis was performed using online tool agriGO. A key on the bottom right indicated the significance of GO terms and their association. (d) Highly connected nodes (hubs) within the co‐expression network are shown. Average degree of turquoise, blue and black modules are higher than the entire co‐expression network (9.936061381). (e) Clustering coefficient (degree to which a node is connected in a neighbourhood) for genes within each node in a box plot is illustrated. Turquoise, blue and black as well as two additional modules (pink and black) display significantly high average clustering coefficient than whole co‐expression network (0.580615941). (f) The information centrality (the flow of information between any two nodes in a connected network) for the largest component of co‐expression network (588 nodes) is presented. Turquoise, blue and black as well as pink modules display significantly increased information centrality compared to the entire network (0.00126154).

With the availability of large‐scale transcriptome data sets, weighted gene co‐expression network analysis (WGCNA) has allowed to identify a cohort of genes with similar expression patterns in response to a given stimulus or physiological condition within a cell (Garbutt *et al*., 2014; Tully *et al*., 2014). Thus, co‐expression networks allow identification of a set of genes which might participate in a common biological process (Smakowska‐Luzan *et al*., 2018). To determine CLCuD‐responsive common gene signatures, we performed WGCNA (Langfelder and Horvath, 2008; Zhang and Horvath, 2005). To construct a co‐expression network, we processed transcripts with FPKM count ≥ 15 and removed all the outliers, which yielded a total number of 1676 DEGs. By using WGCNA platform, we created topological overlap mapping metric (TOM) plot, a measure of neighbourhood proximity that calculates the similarity matrix of genes expression between two nodes (Langfelder and Horvath, 2007). TOM also features hierarchical clustering dendrograms possessing a range of weighted correlations (Langfelder and Horvath, 2008). These analyses led us to generate an undirected weighted network with scale‐free topology, a network with power‐law degree distribution. This weighted co‐expression network encompasses nine different modules (Figure 4a and b; Table S6) that are decorated with nine different colours. Among them, the largest (turquoise) and the smallest (black and pink) modules comprise 455 and 72 genes, respectively.

Centrality measures can reveal the most influential vertices in a network. To decipher the most important nodes within this co‐expression network, we calculated degree (number of connections of a node) and clustering coefficient (degree to which a node is connected in a neighbourhood) as well as information centrality (the flow of information between any two nodes in a connected network) (Seebacher and Gavin, 2011; Tully *et al*., 2014; Wang *et al*., 2011). Degree distribution revealed a total of 52 hubs, nodes with ≥25 connections within the co‐expression network (Figure 4b and Table S5). Furthermore, we determined that average degree of turquoise, blue and black modules are higher than the entire co‐expression network. Intriguingly, we also found that turquoise, blue and black as well as two additional modules (pink and black) exhibit significantly heightened average clustering coefficient (Figure 4d and e). Our information centrality measures in the largest component; that is, 588 nodes determine that turquoise, blue and black as well as pink modules display significantly increased information centrality compared to the entire network (Figure 4f).

### Functional regulation of gene modules reveals disease resistance pathways in Mac7

After the identification of differentially regulated gene modules, we performed gene ontology (GO) analysis on the gene IDs associated with the most interacting 52 hubs (Table S7). The results indicated the association of gene IDs with two major GO categories; localization and response to stimulus, that was further narrowed down to response to auxin stimulus (Figure 4c). Geminiviruses are known to activate the expression of auxin‐inducible genes and interact with the auxin pathway to promote cell proliferation and modulate differentiation in plants (Park *et al*., 2004). Differential regulation of auxin pathway in Mac7 in response to CLCuD might result from TrAP‐mediated inhibition of adenosine kinase, which phosphorylates and converts them to their low‐activity nucleotide forms (Wang *et al*., 2003).

Gene ontology analysis of individual modules was also performed to estimate the functional regulation in each module (Figure S6–S12). The largest module (turquoise; Figure S11) was associated with response to stimulus and ribonucleoprotein complex biogenesis, followed by two modules significantly associated with ribosome biogenesis (Figures S7 and S9). Interestingly, the module consisting of only 42 genes provided the most complex GO term association including regulation of defence response and interaction with host (Figure S8).

### Virus‐induced gene silencing in Mac7 cotton

To demonstrate the direct involvement of CLCuD‐associated genes in disease resistance, we selected three Mac7‐related DEGs for downstream genetics and functional analyses. Heat shock cognate protein 80 (HSC80), that belongs to the heat shock protein 90 (*HSP90*) family, has been reported to enhance the accumulation of geminivirus coat protein in the infected cells (Moshe *et al*., 2016) and overall plays a major role in the establishment of geminivirus infection (Gorovits and Czosnek, 2017). E3 ligase, a RING finger protein, is induced by geminivirus protein C4, affects geminivirus infection by regulating the host cell cycle (Lai *et al*., 2009), and is responsible for ubiquitination and proteasomal degradation of a betasatellite protein βC1(Shen *et al*., 2016). Serine/threonine‐protein kinases have been shown to interact with several geminivirus‐betasatellite proteins (Table 1). Importantly, they interact with βC1 protein and reduce geminivirus DNA accumulation in plants (Shen *et al*., 2012); probably by phosphorylating βC1 (Shen *et al*., 2011). Based on these properties HSC80, E3 ligase and a ser‐thr protein kinase were selected for VIGS analysis. These genes were amplified from Mac7 genomic DNA, cloned in TRV2 VIGS vector and agro‐inoculated in Mac7 (Figure 6a). Among agro‐inoculated Mac7 plants observed 12 days postinoculation, TRV:GrCLA plants showed complete photobleaching (Figure 6b). At that stage, Mac7 plants silenced with target genes were infested with whiteflies. RT‐PCR showed the down‐regulation of target genes in silenced plants compared to TRV:00 plants, 2 weeks postinfestation (Figure 6c). Eggs and pupae were found significantly higher (63–105 eggs/pupa/cm^2^ leaf) on TRV:STK and TRV:HSC80 as compared to control TRV:00 plants (Figure 6d,g). Adult whiteflies were detected to be higher in silenced plants; however, the adults were significantly increased (49–65 adult whiteflies per plant) on TRV: STK, TRV:E3 and TRV:HSC80 plants (Figure 6e). Semi‐quantitative PCR on gDNA shows a minute virus titre in TRV:STK, TRV:E3 ligase and TRV:HSC80 plants; however, no virus titre was detected in TRV:00 plants (Figure 6f).

**Table 1:** Geminivirus‐interacting genes and their regulation in Mac7

Process	Host partner	Viral partner#	Function	Significance in Mac7	Reference
Transcription	SlNAC1/ATF1	Ren	Nac transcription factor	Down	Selth *et al*. (2005)
	JDK	TrAP/C2	Transcription factor	Down	Lozano‐Duran *et al*. (2011)
	CYCD1;1		Cyclin subunit of CD	Down	Ascencio‐Ibanez *et al*. (2008)
	CYCD3;1	RBR	Transcription factor	Up	Ascencio‐Ibanez *et al*. (2008)
	RKP	ICK/KRP	RING finger SPRY domain protein	Down	Lai *et al*. (2009)
	SET7/9	TrAP/C2	H3K4 methyltransferase	Down	Lozano‐Duran *et al*. (2011)
	NIG	NSP	Transport GTPase	Down	Carvalho *et al*. (2008a)
	cpHSC70	MP	Plastid heat shock protein	Up	Lozano‐Duran *et al*. (2011)
	HSC70		Heat shock protein cognate 70	Up	Lozano‐Duran *et al*. (2011)
	rpl10	NIK	Ribosomal protein 10, NIK inhibitor	Up	Carvalho *et al*. (2008b); Rocha *et al*. (2008)
	NsAK	NSP	PERK‐like receptor‐like kinase, wound‐induced	Down	Florentino *et al*. (2006)
	BAM1	C4	CLAVATA1‐related receptor‐like kinase	Down	Lozano‐Duran *et al*. (2011)
	LRR‐RLK	C4		Down	Piroux *et al*. (2007)
	SnRK1	TrAP/C2/BetaC1	SNF1‐related kinase 1	Down	Hao *et al*. (2003)
	SnRK2.1		SNF1‐related kinase 2	Down	Lozano‐Duran *et al*. (2011)
	AtSK2/SlSK	C4	Shaggy‐related kinase, meristem organization	Down	Dogra *et al*. (2009); Lozano‐Duran *et al*. (2011); Piroux *et al*. (2007)
Protein metabolism	ATJ3	REn	Co‐chaperone	Up	Lozano‐Duran *et al*. (2011)
	UBA1	TrAP/C2	Ubiquitin activating enzyme	Down	Lozano‐Duran *et al*. (2011)
	UBC3	BetaC1	Ubiquitin activating enzyme	Down	Eini *et al*. (2009)
	RHF2A		RING‐type E3 ubiquitin ligase	Down	Lozano‐Duran *et al*. (2011)
Silencing/defence	ADK	TrAP/C2	DNA methylation; cytokinin response	Up	Baliji *et al*. (2010); Buchmann *et al*. (2009); Wang *et al*. (2005)
	SAHH	BetaC1	DNA methylation	Up	Yang *et al*. (2011)
	GDU1,3		Glutamine transport, SA pathway	Down	Chen *et al*. (2010)
	PR1		Pathogenesis related protein, SA pathway	Down	Ascencio‐Ibanez *et al*. (2008)
	ACD6		Regulator of salycilic acid pathway	Down	Yang *et al*. (2013)
	GSTF14		Glutathione‐S‐transferase	Down	Yang *et al*. (2013)
	SKL2	CP	Shikimate kinase	Up	Lozano‐Duran *et al*. (2011)
	AT4CL1		4‐coumarate:CoA ligase	Down	Lozano‐Duran *et al*. (2011)
	AOC1		Allene oxide cyclase	Down	Lozano‐Duran *et al*. (2011)
Stress	F14P1.1	C4	Wounding induced	Down	Lozano‐Duran *et al*. (2011)
	RD21	V2	Dehydration responsive	Down	Lozano‐Duran *et al*. (2011)
	PLP2		Patatin‐like protein 2	Down	Lozano‐Duran *et al*. (2011)
Transmission	GroEL	CP	Protein chaperone	Up	Morin *et al*. (1999)

Metabolic changes induced by ser/thr kinases in plants are considered to be important in plant defence against viruses (Hao *et al*., 2003). For instance, a ser/thr kinase SlSnRK1 interacts with betasatellite protein βC1 (Shen *et al*., 2011), phosphoralates βC1 and attenuates geminivirus infection (Shen *et al*., 2012). A proline‐rich extensin‐like receptor protein kinase (PERK), having a C‐terminal ser/thr kinase domain, interacts with geminivirus nuclear‐shuttle protein (NSP) and effects the efficiency of geminivirus infection (Florentino *et al*., 2006). The induction of CLCuD upon attenuation of ser/thr kinase in our VIGS experiment (Figure 6) indicates its importance as a defence protein against CLCuD.

Research has shown that RING‐finger proteins that function as a E3 ubiquitin ligase are involved in plant defence responses (Alcaide‐Loridan and Jupin, 2012; Liu *et al*., 2014), and in suppression of innate immune responses (Citovsky *et al*., 2009; Marino *et al*., 2012). RING‐finger E3 ubiquitin ligases have been shown to interact with betasatellite protein βC1 (Table 1). They can mediate βC1 ubiquitination and attenuate geminivirus symptoms via promoting βC1 degradation by the 26S proteasome (Shen *et al*., 2016). Apart from interacting with βC1, RING finger protein (RKP) also interacts with geminivirus protein C4 and induces plant cell proliferation by targeting cyclin kinase inhibitors for proteosomal degradation (Lai *et al*., 2009). Consistent to what we observed in our experiment (Figure 6), RKP has also been associated with increased virus levels upon geminivirus infection (Lai *et al*., 2009).

*HSP90*, and other related *HSP*s, made a major component of significantly up‐regulated DEGs in Mac7 (Tables S10 and S3), indicating the involvement of *HSP*s in CLCuD resistance in Mac7. The results from our KEGG pathway analysis (Kanehisa *et al*., 2017) indicated that up‐regulation of *HSP*s is involved in the protein recognition and ER‐associated degradation during protein progression in endoplasmic reticulum (Figure S13). In geminivirus‐infected plants, *HSPs* have been shown to be associate with the circulative transmission of viruses in insect (whitefly) vector (Gorovits and Czosnek, 2017). *HSP70* plays an important role in the nuclear CP transportation and replication of begomoviruses. Up‐regulation of *HSP70* in Mac7 might be associated with better host protein protection during CLCuD infection. On the other hand, silencing of *HSP90* leads to enhanced accumulation of begomovirus CP as infection develops (Moshe *et al*., 2016) and its up‐regulation in Mac7 may be associated to the limited CP production and in turn suppression of virus levels. We also observed that *HSP90*‐RAR1 complex, involved in plants hypersensitive response (HR), is up‐regulated in Mac7 (Figure S14). Previous work has shown the induction of HR in plants upon CLCuD, and this might have a major role in CLCuD resistance of Mac7 (Mubin *et al*., 2010). Interestingly, several genes involved in plant–pathogen interaction pathway were observed to be down‐regulated (Figure S14), including a pathogenesis related 1 (*PR1*), which has been demonstrated to induce broad‐spectrum pathogen resistance in cotton (Parkhi *et al*., 2010). This could be a result of viral counter defence mechanism that is triggered as a result of plant defence mechanism against viral pathogens (Pumplin and Voinnet, 2013). Collectively, these results indicated the differential regulation of important biological processes lead to the CLCuD resistance in Mac7.

### Geminivirus‐/betasatellite‐interacting genes are differentially regulated in Mac7

The genome of geminiviruses consists of only six protein‐coding genes, and the associated alpha/betasatellites have only one gene per component. Therefore, geminiviruses have evolved very sophisticated ways of hijacking host cellular machinery and utilizing it for replication and transcription of viral genes (Hanley‐Bowdoin *et al*., 2013). Proteins produced by geminiviruses and satellites interact with several host proteins and alter cellular functions. To evaluate the regulation of geminivirus‐ and betasatellite‐interacting genes, we analysed a comprehensive set of host genes that have been reported to interact with geminiviruses and betasatellites (Hanley‐Bowdoin *et al*., 2013). We then looked for the host gene homologues in *G. hirsutum* and evaluated their differential regulation in Mac7. Results indicated the differential regulation of several geminivirus‐/betasatellite‐interacting genes in Mac7 (Table 1). S‐adenosyl homocysteine hydrolase (SAHH), a methyl cycle enzyme that interacts with betaC1 and is required for transcriptional gene silencing by DNA methylation (Yang *et al*., 2011), was activated. On the other hand, an important ubiquitin activating enzyme UBC3, which interacts with CLCuD betasatellite's betaC1, and this interaction is essential for betasatellite specific symptoms on cotton (Eini *et al*., 2009), was suppressed. Down‐regulation of UBC3 might be linked to the absence of betasatellite and in turn asymptomatic phenotype of Mac7. Geminivirus‐interacting proteins NAC1, RKP, NIG and SK are down‐regulated, where NIKs and RPL10 are up‐regulated in Mac7 (Data S1 discussion). RLK‐related pathway provides defence against CLCuVs and can also be associated with the asymptomatic phenotype of Mac7. These analyses indicated that CLCuD resistance in Mac7 is regulated at multiple molecular checkpoints.

### Disease‐related genes in Mac7 are not subgenome A or D specific

**Figure 5:**
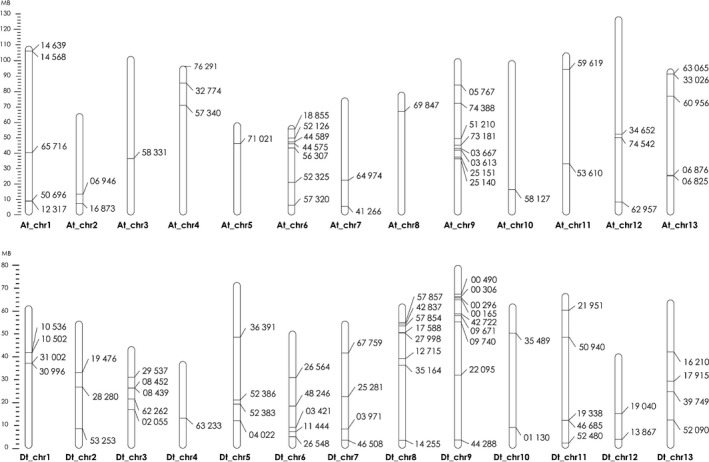
Mapping of disease responsive genes in Mac7 on *Gossypium hirsutum* chromosomes. Chromosomes of subgenome A are indicated with At_ and D with Dt_. A ladder on the left indicates the length of chromosomes and distance between genes in mega bases (MB). Markings on each chromosome represent the respective CLCuD responsive genes in Mac7, and the corresponding numbers represent the gene IDs. The map indicates the clear even distribution of disease responsive genes among both subgenomes.

Cultivated cotton (*G. hirsutum*) is an allotetraploid species and originated as a result of interspecific hybridization between a *G. arboreum*‐like A genome and *G. raimondii*‐like D genome (Paterson, 2008). Diploid progenitor species *G. arboreum* is naturally immune to CLCuD, and we have recently started to understand the underlying mechanisms of this resistance (Naqvi *et al*., 2017). Therefore, we speculated that in Mac7, the CLCuD resistance‐responsive genes may be subgenome A‐specific. To evaluate this, we generated a map by placing the identified DEGs on respective locations on *G. hirsutum* chromosomes. The results indicated that the DEGs identified in this study are not subgenome A‐ or D‐specific and are evenly originated from both subgenomes (Figure 5). *Gossypium raimondii* as a source of CLCuD resistance has never been evaluated, and the origin of several resistance genes from D subgenome indicates that *G. raimondii* may also serve as a source of CLCuD resistance. Moreover, we observed some hotspots for resistance‐responsive genes, for example on chromosomes At_Chr6, At_Chr9, Dt_Chr8 and Dt_Chr9 (Figure 5). Further breeding experiments on Mac7 will help fine mapping these disease resistance loci.

In conclusion, this study provides the molecular insight into an exotic source of CLCuD‐resistant *G. hirsutum*, accession Mac7. The absence of CLCuD symptoms can be directly associated with attenuation of betasatellite in Mac7. We report nine novel modules associated with defence response to CLCuD in Mac7. Multiple disease resistance mechanisms and geminivirus‐/betasatellite‐interacting genes are differentially regulated in Mac7, and disease resistance genes in Mac7 are not originating from a specific A or D subgenome. This study provides a high‐quality transcriptomic data set associated with geminivirus disease resistance, which will be a very helpful tool for future experiments on understanding plant–geminivirus interaction and developing durable CLCuD resistance.

## Experimental procedures

### Screening of Mac7 for CLCuD resistance

Mac7 is a *G. hirsutum* accession and was obtained for this study under ‘Pakistan‐U.S. Cotton Productivity Enhancement Program’ of ‘International Center for Agricultural Research in the Dry Areas (ICARDA)’ from the United States Department of Agriculture (USDA), Stoneville, Mississippi (ARS P.0063.14), USA. Plants of Mac7 were grown in two separate glass houses. In first glasshouse, whitefly‐free conditions were maintained and in second glasshouse, plants were infested with CLCuD viruliferous whiteflies. In the second glasshouse, a CLCuD susceptible *G. hirsutum* variety ‘Karishma’ was also grown at the same time, to be monitored as control plants for CLCuD symptoms. The plants were exposed to whiteflies 6 weeks post germination. Regarding the source of whiteflies, the whiteflies were collected from cotton fields and maintained at the green house on plants of *G. hirsutum* variety Karishma. The whiteflies maintained at the green house were analysed and confirmed to be *B. tabaci* Asia II 1, the most important whitefly biotype that played a significant role in epidemiology of CLCuD (Masood *et al*., 2017; Pan *et al*., 2018). We also collected CLCuD‐symptomatic cotton samples from field and analysed them for begomovirus diversity. The results indicated the presence of begomovirus–satellite complex that we have discussed in another study (Zubair *et al*., 2017b). Further, we also confirmed the presence of begomovirus and betasatellites by PCR using universal begomovirus primers (Shahid *et al*., 2007) and betasatellite primers (Briddon *et al*., 2002). Thus, the PCR confirmation and the induction of severe CLCuD symptoms on CLCuD‐susceptible karishma plants were indicative of the viruliferous whiteflies. The temperature of the glasshouse was maintained between 38 and 45 °C during the day and 25–30 °C for night‐time (optimal for CLCuD‐whitefly infection). Leaf tissue was collected from CLCuD symptomatic and asymptomatic plants at day 25 postinfestation (Figure 1), and genomic DNA was extracted with the CTAB method (Doyle and Doyle, 1989).

### RNA extraction, library construction and RNA‐Seq

A schematic workflow of RNA‐Seq methodology adopted in this study is represented in Figure 2a. Total RNA was extracted from control and CLCuD‐infested leaves of Mac7 using TRIzol reagent according to the manufacturer's instructions (Invitrogen, Carlsbad, CA). Four biological replicates from each sample were used for this experiment. The quality and quantity of RNA were assessed by electrophoresis on 1% agarose gels and by a NanoDrop 1000 spectrophotometer (Thermo Fisher Scientific, Waltham, MA). The integrity of RNA samples was examined using Bio‐analyzer 2100 equipment (Agilent Technologies, Ratingen, Germany). The extracted total RNA samples with a concentration of 10 μg were used for cDNA synthesis, and strand specific RNA‐Seq libraries were constructed as described earlier (Zhong *et al*., 2011). Poly (A) mRNA was isolated using oligo‐dT beads (Qiagen, Hilden, Germany). The mRNA was broken into short fragments (~300 nt). First‐strand cDNA was synthesized using random hexamer‐primed reverse transcription. Second‐strand cDNA was generated using RNase H and DNA polymerase I. The cDNA fragments were purified and washed for end repair and ligated to sequencing adapters. The cDNA fragments of suitable size were purified and enriched by PCR to obtain the final cDNA library. The integrity of each cDNA library was examined using Bio‐analyzer 2100 equipment (Agilent Technologies, Germany). The cDNA libraries were then sequenced using single‐end mode of HiSeq^™^ 2500 equipment (Illumina, San Diego, CA) that yielded SE reads with an average 150 read length. RNA‐Seq data in this study have been deposited at the NCBI under the BioProject ID PRJNA390823.

### RNA‐Seq data analysis

Cleaned reads were selected after preprocessing with Trimmomatic (Bolger *et al*., 2014) to remove low‐quality sequences (i.e. reads containing adaptor sequences, and reads with more than 5% unknown bases). Quality of individual sequences was evaluated using FastQC (Andrews, 2010), including per base sequence quality analysis which plots the Phred quality score distribution for each read generated per sample for each nucleotide base call (Figure S1). All FASTQ sequencing files obtained in this study have an average per base Phred score of 37, a conventional threshold denoting high‐quality base calls (Figure S1 and Table S1). The cleaned reads were then mapped to the reference *G. hirsutum* genome (Li *et al*., 2015) using HISAT2 (Pertea *et al*., 2016). Default HISAT2 parameters, which allow up to two mismatches and report up to 20 alignments for reads mapping at multiple positions, were used. The sequence alignment/map files generated by HISAT2 were used as the input to the software Cufflinks (Trapnell *et al*., 2012) which assembles the alignments in the sequence alignment/map file into transfrags. Cufflinks does this assembly independently of the existing gene annotations and constructs a minimum set of transcripts that best describes the RNA‐Seq reads. The unit of measurement used by Cufflinks to estimate transcript abundance is FPKM. The Cufflinks statistical model probabilistically assigns reads to the assembled isoforms. Cuffdiff was used to find differentially expressed genes (DEGs). The read coverage of one gene was used to calculate the gene expression level, which was measured with the reads per kilobase of exon model per million mapped reads (RPKM) method. A *q*‐value cut‐off of 0.05 was used to determine whether a gene had differential expression between samples. In the comparison of gene expression levels of control and CLCuD‐infested Mac7 plants, an absolute value of log2 fold change > 1 and the false discovery rate (FDR) < 0.05 was set to declare DEGs involved in the response of CLCuD infestation. Differential gene expression was quantified by comparing the transcriptome of CLCuD‐free Mac7 (RS5‐RS8) with the CLCuD‐infested Mac7 (RS1‐Rs4) using cuffdiff within the cufflinks package (Trapnell *et al*., 2012). Analysis was performed by treating all sequencing files individually, again to ensure the reproducibility of the experiment (Figure 2a). Heatmaps were drawn using heatmap.2 function of the gplots package in R (Figure 2f).

### Gene ontology and pathway analysis

To determine the main biological functions and pathways of the significant DEGs, these were mapped to terms in the Gene Ontology (GO) and Kyoto Encyclopedia of Genes and Genomes (KEGG) databases using KOBAS 3.0 (http://kobas.cbi.pku.edu.cn/index.php). KEGG pathways were manually evaluated for up‐regulation or down‐regulation at respective points. GO terms were annotated and depicted using agriGO (http://bioinfo.cau.edu.cn/agriGO/).

### Construction of co‐expression network and network analyses

To construct a co‐expression network, we processed transcripts with FPKM count ≥ 15 count and removed all the outliers, which yielded a total number of 1676 DEGs. We implemented R‐based Weighted Gene Co‐expression Network Analysis (WGCNA) (Langfelder and Horvath, 2008; Zhang and Horvath, 2005) package to construct a co‐expression network. The dendrogram was constructed using the cutreeDynamicTree algorithm (Zhang and Horvath, 2005), with a threshold of minimum module size of 70 genes. A weighted correlation threshold of ≥0.85 was set that resulted in the identification of nine modules with respective colours presenting the entire network. Python‐based NetworkX (Hagberg *et al*., 2008) and Cytoscape v. 3.5.1 Plugins (Saito *et al*., 2012) were utilized for network analyses including information centrality, degree and cluster coefficients. Weighted co‐expression network was visualized using ‘Group Attributes Layout’, a feature of Cytoscape v. 3.5.1 (Demchak *et al*., 2014; Shannon *et al*., 2003). Box plot was used to display the distribution of nodes within each module for information centrality, degree and cluster coefficients. Student's *t*‐test describes statistical significance.

### Quantitative real‐time PCR

To verify the differential expression detected by the Illumina RNA‐Seq data, quantitative real‐time PCR (qRT‐PCR) was performed on a new set of control and infested samples. A set of 24 genes was chosen (Table S10), including 12 up‐regulated and 12 down‐regulated genes. Primers for qPCR were designed with program Primer3 (http://bioinfo.ut.ee/primer3-0.4.0/primer3) with default settings. All primer sequences are provided in Table S4. qRT‐PCR was performed using a QUANTSTUDIO 6 flex qRT‐PCR instrument and the light cycler fast start DNA Master SYBR Green I kit (Roche, Basel, Switzerland). Reactions were performed in triplicate, and contained 100 ng of cDNA, 0.5 μL of each primer (10 μm/μL), and 10 μL SYBR Green Master Mix in a final volume of 20 μL. The amplification reactions were performed under the following conditions: 95 °C for 5 min, followed by 40 cycles of 95 °C for 15 s, 55 °C for 20 s, and 72 °C for 30 s. Melting curve analysis, performed by increasing the temperature from 55 to 95 °C (0.5 ° per 10 s), and gel electrophoresis of the final product confirmed the presence of single amplicons. Relative fold differences for each sample in each experiment were calculated using the ΔΔ Ct method (Reference). The 18S/Ubiquitin gene was used as a control. To corroborate the expression levels measured by RNA‐Seq, the ratio of expression as measured by qRT‐PCR was compared to the ratio of expression levels between samples using RNA‐Seq. qRT‐PCR for detection and quantification of begomovirus and betasatellite was performed following the methodology as described in Shafiq *et al*. (2017) with respective primers as described in Zaidi *et al*. (2016).

### Virus‐induced gene silencing in Mac7 cotton

**Figure 6:**
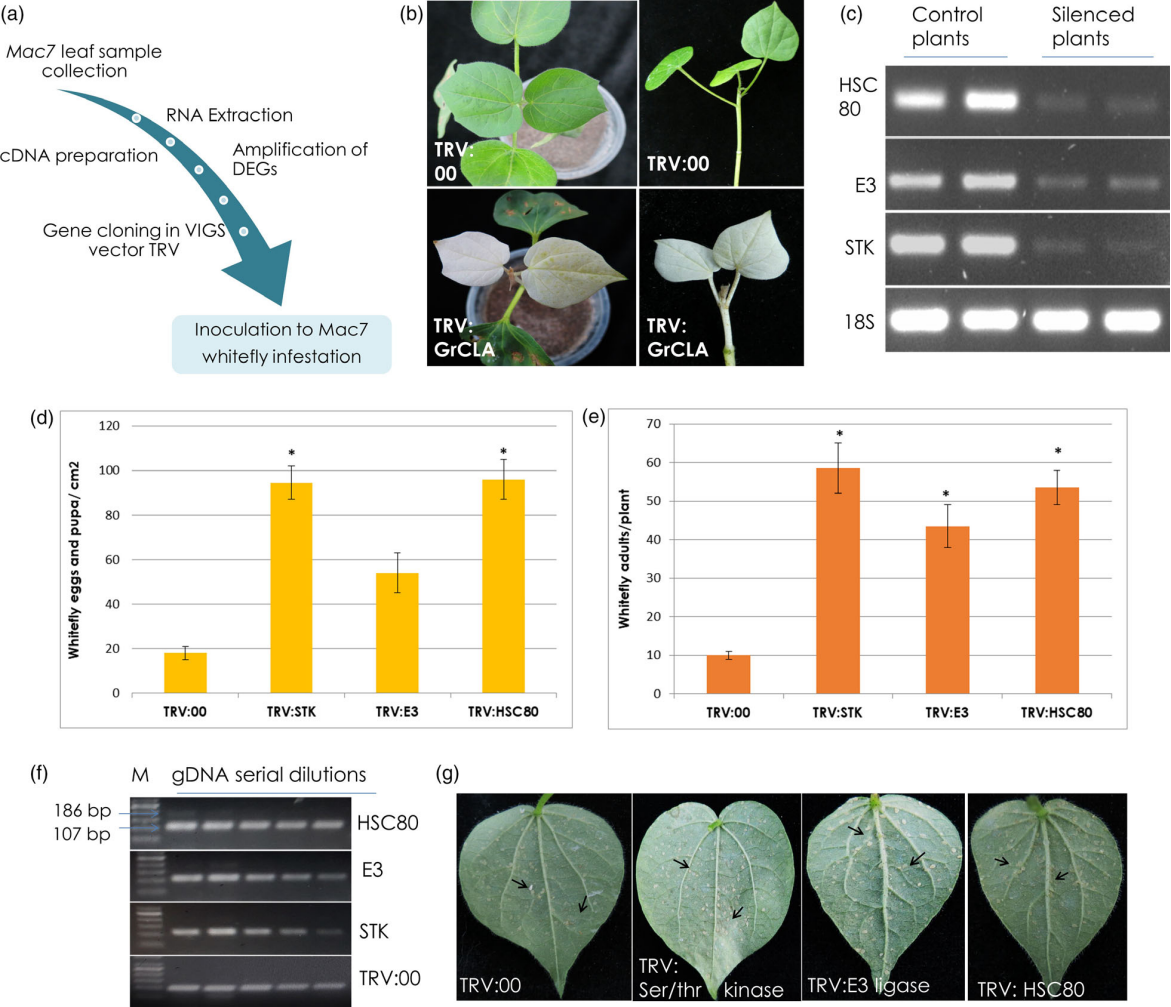
Validation of transcriptomic data by virus‐induced gene silencing of selected genes in Mac7. (a) General methodology adopted for virus‐induced gene silencing (VIGS). Ten‐day old young plantlets of Mac7 were used for agro‐inoculation of plants with VIGS vectors. (b) After 12 days postinoculation, TRV:00 plants showed no change in phenotype, while the bleaching phenotype was observed in TRV:GrCLA plants confirming the efficiency of VIGS system. (c) RT‐PCR showing the down‐regulation of respective genes in silenced plants compared to TRV:00 plants. (d) Quantification of whitefly eggs and pupa on Mac7 VIGS plants 2 weeks post‐whitefly infestation. Error bars represents standard error among biological replicates, and *shows significance using Student's *t*‐test. (e) Quantification of whitefly adults on Mac7 VIGS plants 2 weeks post‐whitefly infestation. Error bars represents standard error among biological replicates, and *shows significance using Student's *t*‐test. (f) Semi‐quantitative PCR shows a minute virus titre in plants silenced for STK, E3 ligase and HSC80. The lower band of 107 bp with higher intensity indicates the cotton endogenous gene Sad1, and the upper band is 186 bp showing virus presence, M: 50 bp DNA marker. (g) Representative images of Mac7 VIGS plants after 2 weeks of whitefly infestation, and black arrows represent whitely eggs and pupae.

Three candidate genes were selected for virus‐induced gene silencing (VIGS) analysis (Table S9) on the basis of the reported role(s) of candidate genes in geminivirus infection. Target genes were amplified from Mac7 genomic DNA and ligated to VIGS vector TRV2. GrCLA was used as positive control of the experiment to judge the VIGS efficiency. Ten‐day‐old young plantlets of Mac7 were used for agro‐inoculation of plants with these VIGS clones (Figure 6a). The inoculation was done according to the previous methods (Gao and Shan, 2013; Mustafa *et al*., 2016). After 12 days postinoculation, TRV:00 plants showed no change in phenotype, while the bleaching phenotype was observed in TRV:GrCLA plants indicating the efficiency of VIGS system. Based on this observation, two sets of plants (with target genes silenced) were made; one was placed with viruliferous whitefly infestation and other one in control conditions. Plants were maintained, and 2 weeks postinfestation, expression levels of target genes in control and silenced plants were detected by RT‐PCR. We also observed the density of eggs and adult whiteflies on the infested plants. To detect the virus titre, genomic DNA was isolated from VIGS silenced plants and quantitative PCR was run using primers F: ATGTGGGATCCACTGTTAAATGAGTTCCC and R: GATTATATCTGCTGGTCGCTTCGACATAA according to the methodology described earlier (Shafiq *et al*., 2017).

## Conflict of interests

Authors declare no competing financial interests.

## Author contributions

S. S. Zaidi, R. Z. Naqvi, S. Mansoor, J. A. Scheffler, B. E. Scheffler and L. A. Mueller designed research; S. S. Zaidi, R. Z. Naqvi, S. Shakir, M. Shafiq, A. M. Khan and M. Asif performed research; I. Amin contributed new reagents/analytic tools; S. S. Zaidi, R. Z. Naqvi, S. Strickler B. Mishra and S. Mukhtar analysed data; B.E. Scheffler performed the viral sequencing; J. A. Scheffler developed and provided the Mac7 seed; and S. S. Zaidi wrote the first draft. All authors read and approved manuscript.

## Supporting information


**Figure S1** FastQC analysis of all samples used for RNA‐Seq.
**Figure S2** Distribution of Log10FPKM in each condition, CLCuD free q1 and CLCuD infected q2.
**Figure S3** Gene dispersion analysis performed on the RNA‐Seq data using cummeRbund package in R, q1 indicates CLCuD free and q2 indicates CLCuD infected.
**Figure S4** Differentially expressed genes identified in Mac7 and drawn using edgeR package in R.
**Figure S5** qPCR results of comparison of both cotton leaf curl disease components, begomovirus and betasatellite.
**Figure S6** GO terms associated with black module in WGCNA analysis.
**Figure S7** GO terms associated with blue module in WGCNA analysis.
**Figure S8** GO terms associated with brown module in WGCNA analysis.
**Figure S9** GO terms associated with grey module in WGCNA analysis.
**Figure S10** GO terms associated with red module in WGCNA analysis.
**Figure S11** GO terms associated with turquoise module in WGCNA analysis.
**Figure S12** GO terms associated with yellow module in WGCNA analysis.
**Figure S13** Protein processing in endoplasmic reticulum pathway analysis performed using online tool KOBAS. Color key on the top right indicates the downregulation (blue), upregulation (pink) and non‐significant regulation (green) in Mac7 RNA‐Seq data.
**Figure S14** Plant‐pathogen interaction pathway analysis performed using online tool KOBAS. Color key on the top right indicates the downregulation (blue), upregulation (pink) and non‐significant regulation (green) tn Mac7 RNA‐Seq data.
**Table S1** RNA‐Seq samples under each treatment with number of raw and trimmed reads, percent mapping and GC, and average Phred score.
**Table S2** FPKMs of housekeeping genes in RNA‐Seq data with respective gene IDs, *P*‐values and *q*‐values.
**Table S3** Differentially expressed genes in transcriptomic data of CLCUD infested versus disease free Mac7, with respective gene IDs, FPKMs, log2FC, *P*‐values and *q*‐values.
**Table S4** List of primers used in this study for qPCR validation of RNA‐Seq data.
**Table S5** Network analyses for resist mock.
**Table S6** Information centrality analysis.
**Table S7** GO terms associated with highly connected 52 hubs identified in WGCNA, with respective *P* values.
**Table S8** Differentially expressed genes in transcriptomic data of CLCUD‐susceptible cotton line (karishma) versus CLCuD‐resistant Mac‐7, with respective gene IDs and log2FC FPKMs.
**Table S9** Genes selected for virus‐induced gene silencing (VIGS) experiment and their respective primers.
**Table S10** List of differentially expressed genes identified in RNA‐Seq and confirmed with qPCR.
