# Supplementary material for: Molecular insight into cotton leaf curl geminivirus disease resistance in cultivated cotton (Gossypium hirsutum)
Source: Plant Biotechnol J. 2019 Sep 30;18(3):691–706. doi: 10.1111/pbi.13236 (PMC7004920; doi:10.1111/pbi.13236)
Supplement: Supplementary file 1 — Figure S1 FastQC analysis of all samples used for RNA‐Seq. Figure S2 Distribution of Log10FPKM in each condition, CLCuD free q1 and CLCuD infected q2. Figure S3 Gene dispersion analysis performed on the RNA‐Seq data using cummeRbund package in R, q1 indicates CLCuD free and q2 indicates CLCuD infected. Figure S4 Differentially expressed genes identified in Mac7 and drawn using edgeR package in R. Figure S5 qPCR results of comparison of both cotton leaf curl disease components, begomovirus and betasatellite. Figure S6 GO terms associated with black module in WGCNA analysis. Figure S7 GO terms associated with blue module in WGCNA analysis. Figure S8 GO terms associated with brown module in WGCNA analysis. Figure S9 GO terms associated with grey module in WGCNA analysis. Figure S10 GO terms associated with red module in WGCNA analysis. Figure S11 GO terms associated with turquoise module in WGCNA analysis. Figure S12 GO terms associated with yellow module in WGCNA analysis. Figure S13 Protein processing in endoplasmic reticulum pathway analysis performed using online tool KOBAS. Color key on the top right indicates the downregulation (blue), upregulation (pink) and non‐significant regulation (green) in Mac7 RNA‐Seq data. Figure S14 Plant‐pathogen interaction pathway analysis performed using online tool KOBAS. Color key on the top right indicates the downregulation (blue), upregulation (pink) and non‐significant regulation (green) tn Mac7 RNA‐Seq data. Table S1 RNA‐Seq samples under each treatment with number of raw and trimmed reads, percent mapping and GC, and average Phred score. Table S2 FPKMs of housekeeping genes in RNA‐Seq data with respective gene IDs, P‐values and q‐values. Table S4 List of primers used in this study for qPCR validation of RNA‐Seq data. Table S9 Genes selected for virus‐induced gene silencing (VIGS) experiment and their respective primers. Table S10 List of differentially expressed genes identified in RNA‐Seq and confirmed with qPCR. [file PBI-18-691-s005.docx]

**Supplementary Information**

**Molecular insight into cotton leaf curl geminivirus disease resistance in cultivated cotton (*Gossypium hirsutum*)**

Syed Shan-e-Ali Zaidi ^1, 2, 3, †^, Rubab Zahra Naqvi ^1, 2, †^, Muhammad Asif ^1^, Susan Strickler ^2^, Sara Shakir ^1, 2, 3^, Muhammad Shafiq ^1, 4^, Abdul Manan Khan ^1^, Imran Amin ^1^, Bharat Mishra ^5^, M. Shahid Mukhtar ^5^, Brian E. Scheffler ^6^, Jodi A. Scheffler ^6^, Lukas A. Mueller^2^ and Shahid Mansoor^1,*^

**^1^** National Institute for Biotechnology and Genetic Engineering, 38000 Faisalabad, Pakistan

**^2^** Boyce Thompson Institute, 533 Tower Rd., Ithaca, NY 14853, USA

**^3^** Plant Genetics Lab, TERRA Teaching and Research Center, Gembloux Agro-Bio Tech, University of Liège, Gembloux, Belgium

**^4^** Present address: Department of Biotechnology, University of Okara, Okara, Pakistan

**^5^** Department of Biology, University of Alabama at Birmingham, Birmingham, AL 35294, USA

**^6^** Genomics and Bioinformatics Research Unit, United States Department of Agriculture-Agricultural Research Service (USDA-ARS), Stoneville, MS 38776, USA

**^7^** Crop Genetics Research Unit, United States Department of Agriculture-Agricultural Research Service (USDA-ARS), Stoneville, MS 38776, USA

**^†^**These authors contributed equally to this work.

*****Correspondence: Shahid Mansoor ([shahidmansoor7@gmail.com](mailto:shahidmansoor7@gmail.com))

Ph. +923007944841

**Running title:** Molecular insight into cotton leaf curl disease resistance in Mac7

**Keywords:** *Gossypium hirsutum*, leaf curl disease, plant virus resistance, transcriptome, WGCNA

**Supplementary Discussion**

**Suppression of NAC1, RKP, NIG and SK in Mac7**

Geminivirus protein REn interacts with the host NAC1, which is a plant transcription factor that accumulates in virus-positive cells of infected leaves. Ectopic expression of the target gene of NAC1 increases viral DNA levels ([Selth et al., 2005](#_ENREF_11)). The C4 proteins of begomoviruses induces plant cell proliferation by activating expression of a host RING finger protein (RKP), which targets cyclin kinase inhibitors for proteosomal degradation ([Lai et al., 2009](#_ENREF_6)). Upon begomovirus infection, the induction of RKP is associated with increased virus levels ([Lai et al., 2009](#_ENREF_6)). An NSP-interacting GTPase (NIG) associated with the exterior of the nuclear envelope might facilitate NSP transit into the cytosol, probably through the nuclear pore ([Carvalho et al., 2008a](#_ENREF_1)). The NSP–DNA complex then moves to the cell periphery through interaction with MP ([Sanderfoot and Lazarowitz, 1995](#_ENREF_10)). Viral DNA might be transferred to MP through a mechanism involving NIG-catalyzed GTP hydrolysis ([Carvalho et al., 2008b](#_ENREF_2)). Alternatively, NIG might facilitate the interaction of MP with an NSP-DNA complex that moves through plasmodesmata, which provides a mechanism for movement of viral DNA into the nucleus of the next cell. Shaggy-related kinases (SK) are involved in various plant developmental processes, including cell division and elongation, in part through their interactions with the brassinosteroid signaling pathway. Geminivirus C4 protein interacts with SK, and silencing the expression of SK delays infection ([Dogra et al., 2009](#_ENREF_4); [Lozano-Duran et al., 2011](#_ENREF_7); [Piroux et al., 2007](#_ENREF_8)). A common feature among of NAC1, RKP, NIG and SK is that they interact with geminivirus proteins and enhance virus levels. Their downregulation in Mac7 might be linked to the decreased virus levels in Mac7 (Table 2).

**Induction of defense related RLKs in Mac7**

Some plant receptor-like kinases (RLKs) sense viral pathogens and trigger an antiviral defense response. The best characterized RLKs involved in geminivirus infection are the three closely related leucine- rich repeat (LRR) RLKs designated NSP-interacting kinase 1 (NIK1), NIK2 and NIK3 ([Fontes et al., 2004](#_ENREF_5)). Geminivirus NSP binds to the NIK kinase domain and interferes with its autophosphorylation, which is required for kinase activity ([Fontes et al., 2004](#_ENREF_5)). NIK proteins are thus unable to phosphorylate their downstream effector, the ribosomal protein RPL10, and induce its translocation to the nucleus, where it interferes with viral infection ([Rocha et al., 2008](#_ENREF_9)). The activities of NIK proteins and RPL10 correlate with symptom development; overexpression of these proteins attenuates and delays symptoms, whereas loss of their function increases susceptibility ([Carvalho et al., 2008c](#_ENREF_3)). Upregulation of both NIKs and RPL10 in Mac7 (Table 2) suggests that RLK related pathway provides defense against invading virus and thus, in combination with other pathways, Mac7 plant remains asymptomatic for CLCuD. In light of our data analyses and above discussion, it can be concluded that Mac7 used this multiple layered defense response to trigger resistance against CLCuD.

**Supplementary Figures**

**
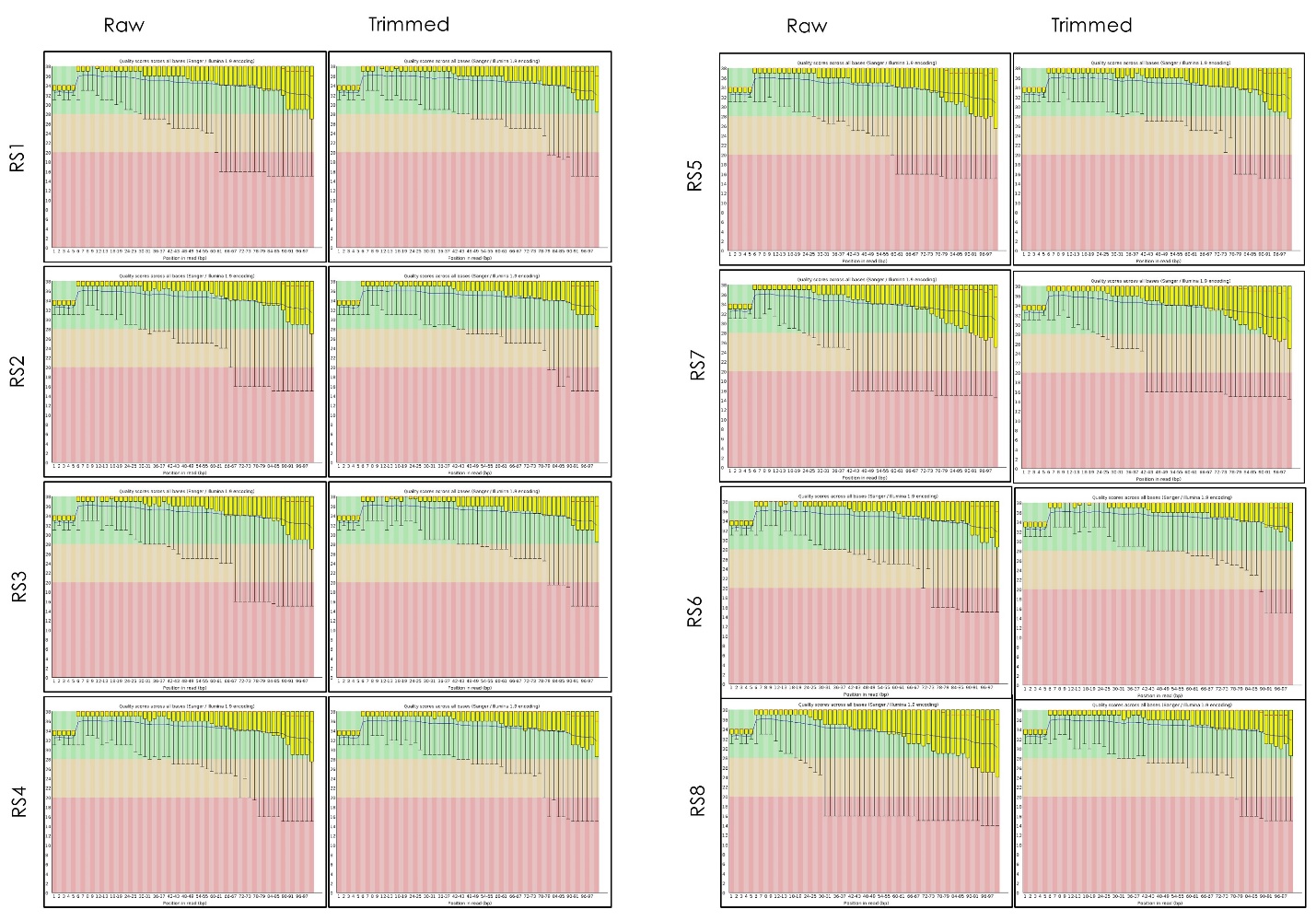
Figure S1.** FastQC analysis of all samples used for RNA-Seq

**
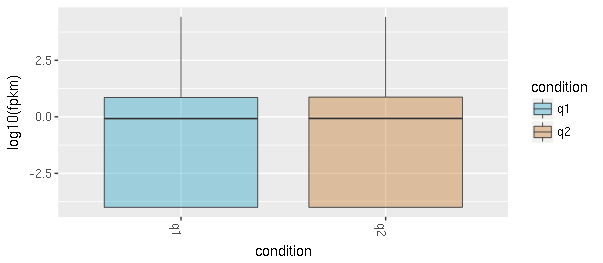
Figure S2.** Distribution of Log10FPKM in each condition, CLCuD free q1 and CLCuD infected q2.

**
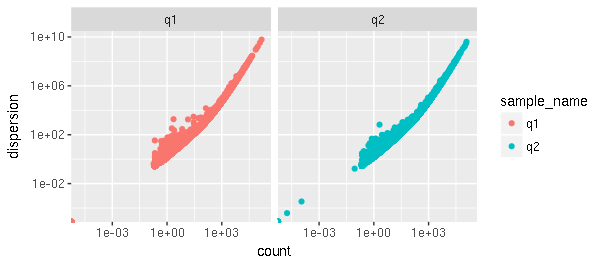
Figure S3.** Gene dispersion analysis performed on the RNA-Seq data using cummeRbund package in R, q1 indicates CLCuD free and q2 indicates CLCuD infected.

**
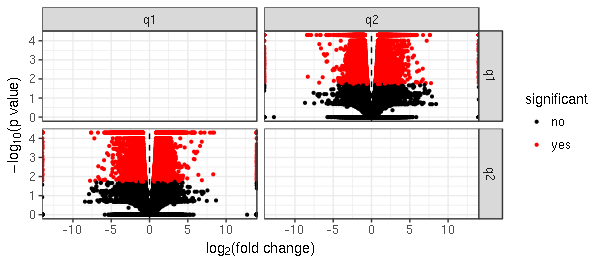
Figure S4.** Differentially expressed genes identified in Mac7 and drawn using edgeR package in R

**
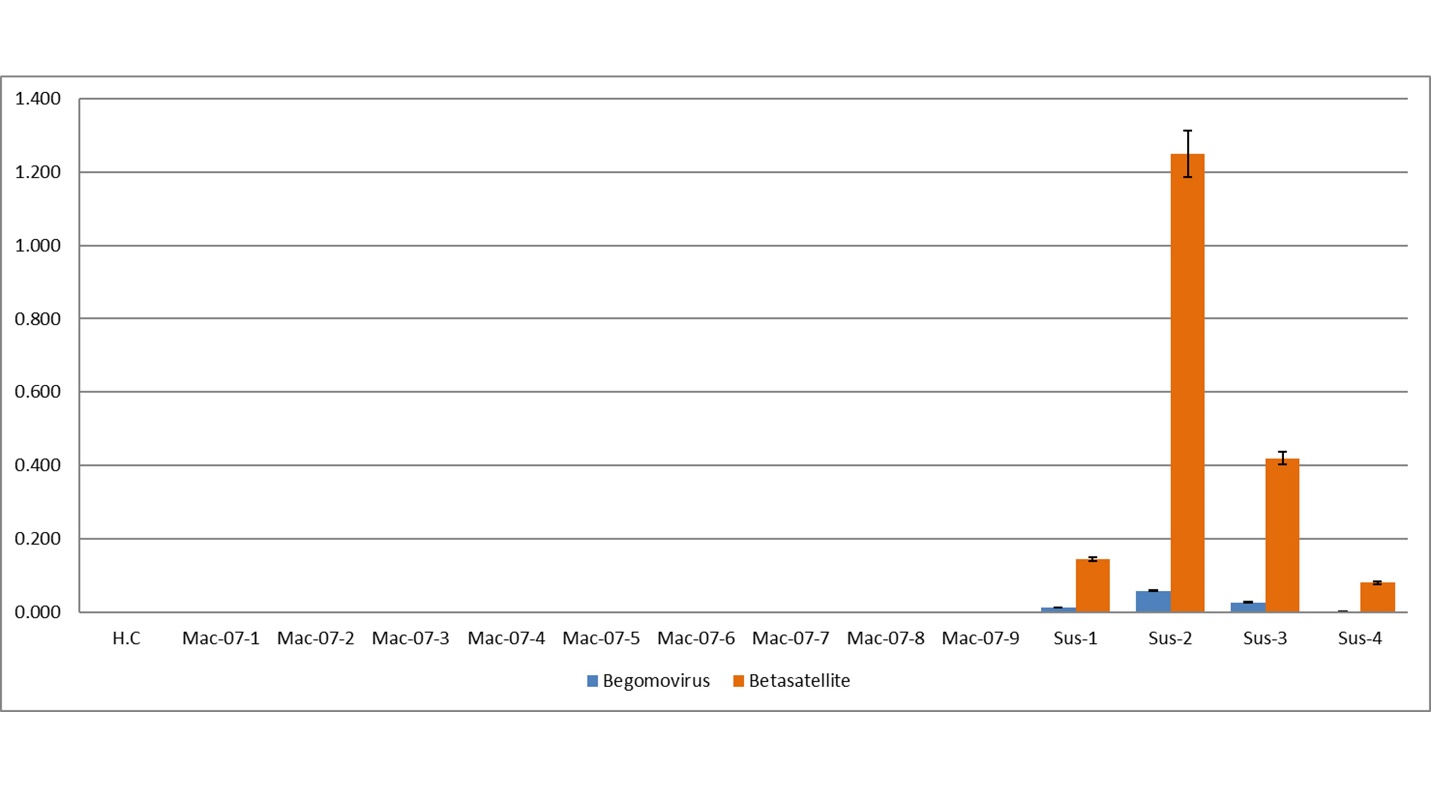
**

**Figure S5.** qPCR results of comparison of both cotton leaf curl disease components, begomovirus and betasatellite.

**
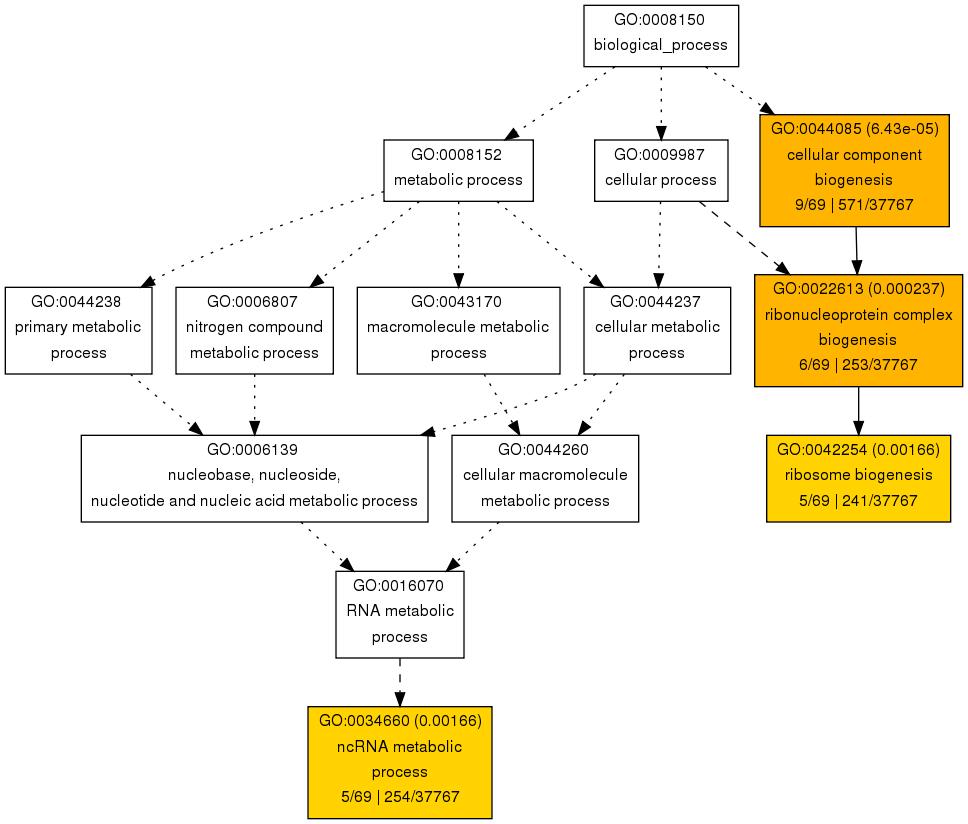
Figure S6.** GO terms associated with black module in WGCNA analysis.

**
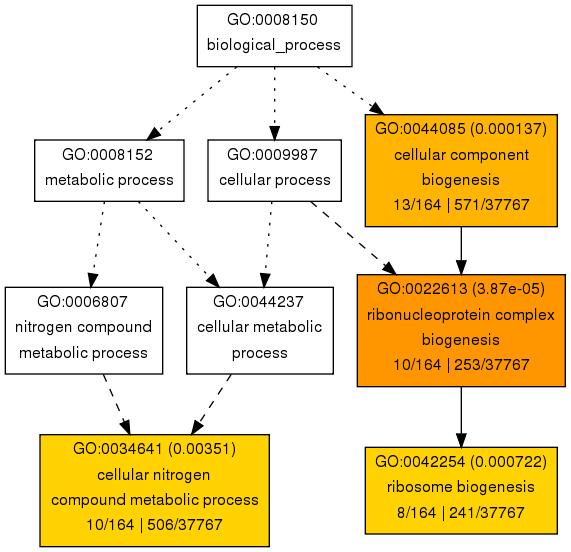
**

**Figure S7.** GO terms associated with blue module in WGCNA analysis.

**
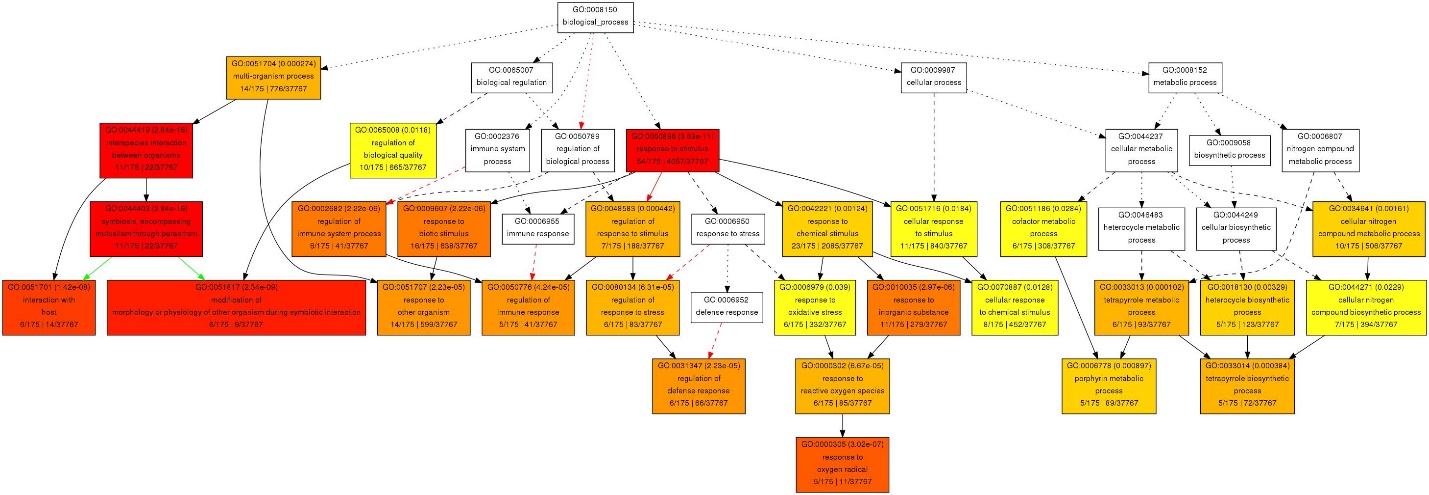
Figure S8.** GO terms associated with brown module in WGCNA analysis.

**
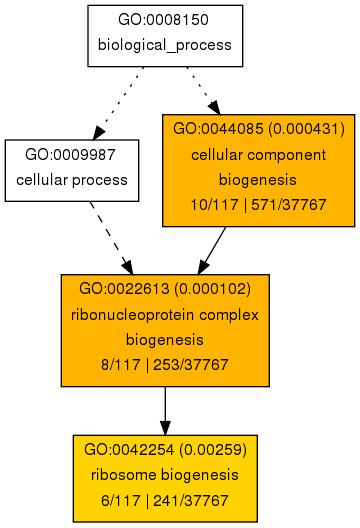
**

**Figure S9.** GO terms associated with grey module in WGCNA analysis.


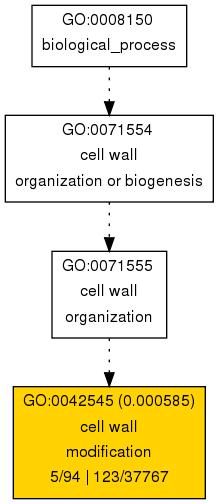


**Figure S10.** GO terms associated with red module in WGCNA analysis.


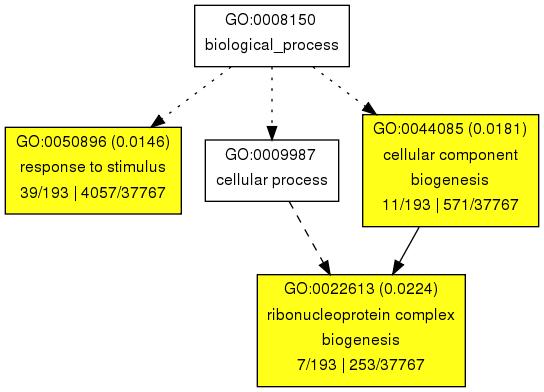


**Figure S11.** GO terms associated with turquoise module in WGCNA analysis.

**
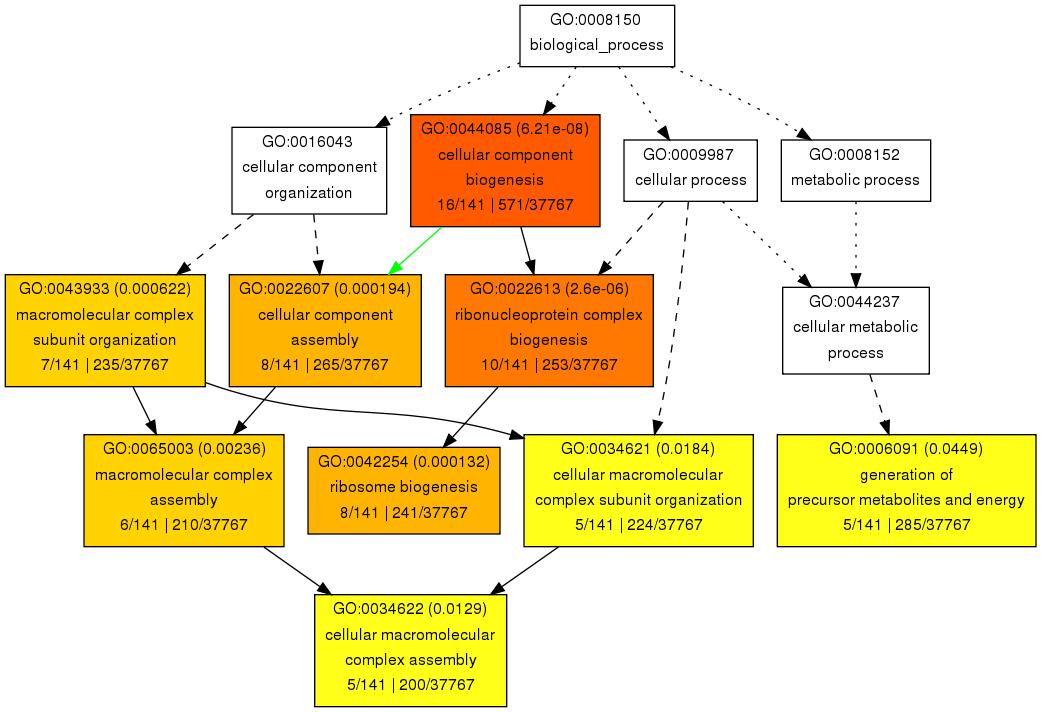
**

**Figure S12.** GO terms associated with yellow module in WGCNA analysis.


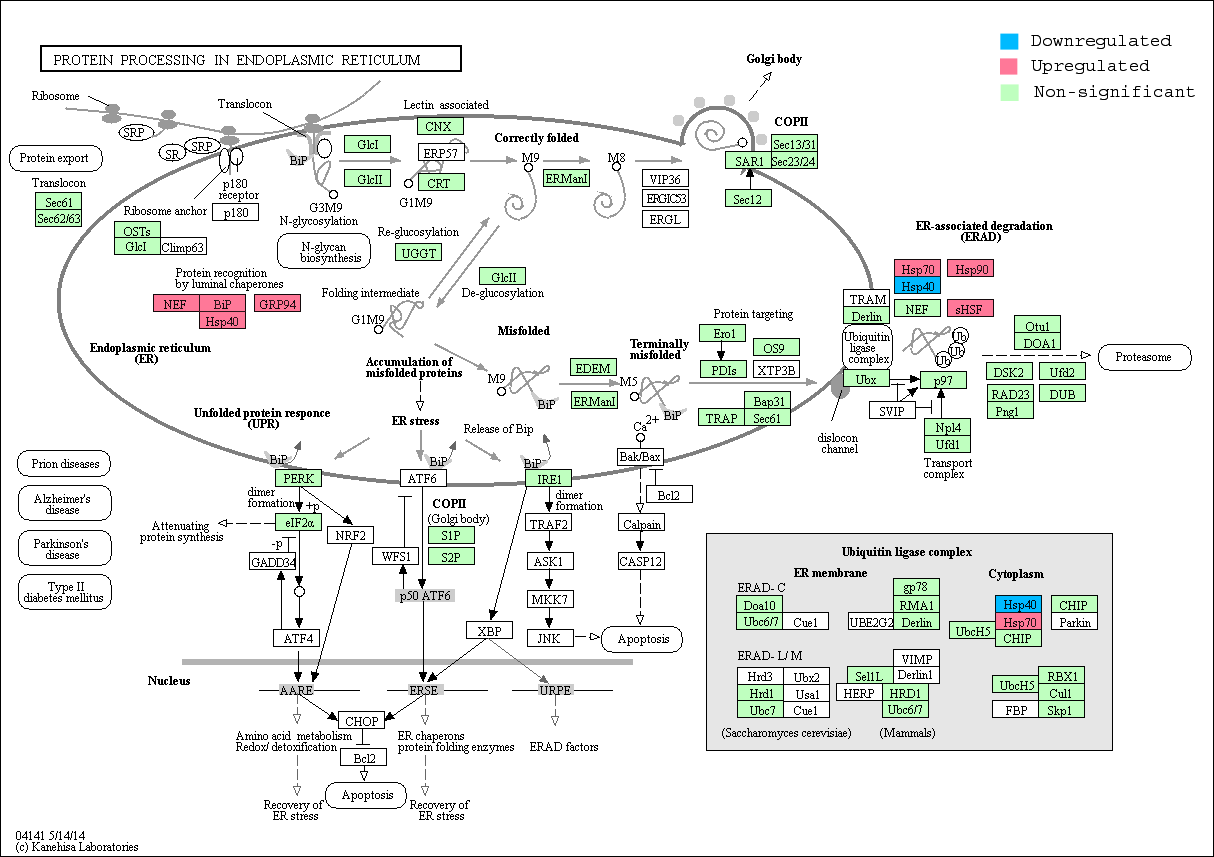


**Figure S13.** Protein processing in endoplasmic reticulum pathway analysis performed using online tool KOBAS. Color key on the top right indicates the downregulation (blue), upregulation (pink) and non-significant regulation (green) in Mac7 RNA-Seq data


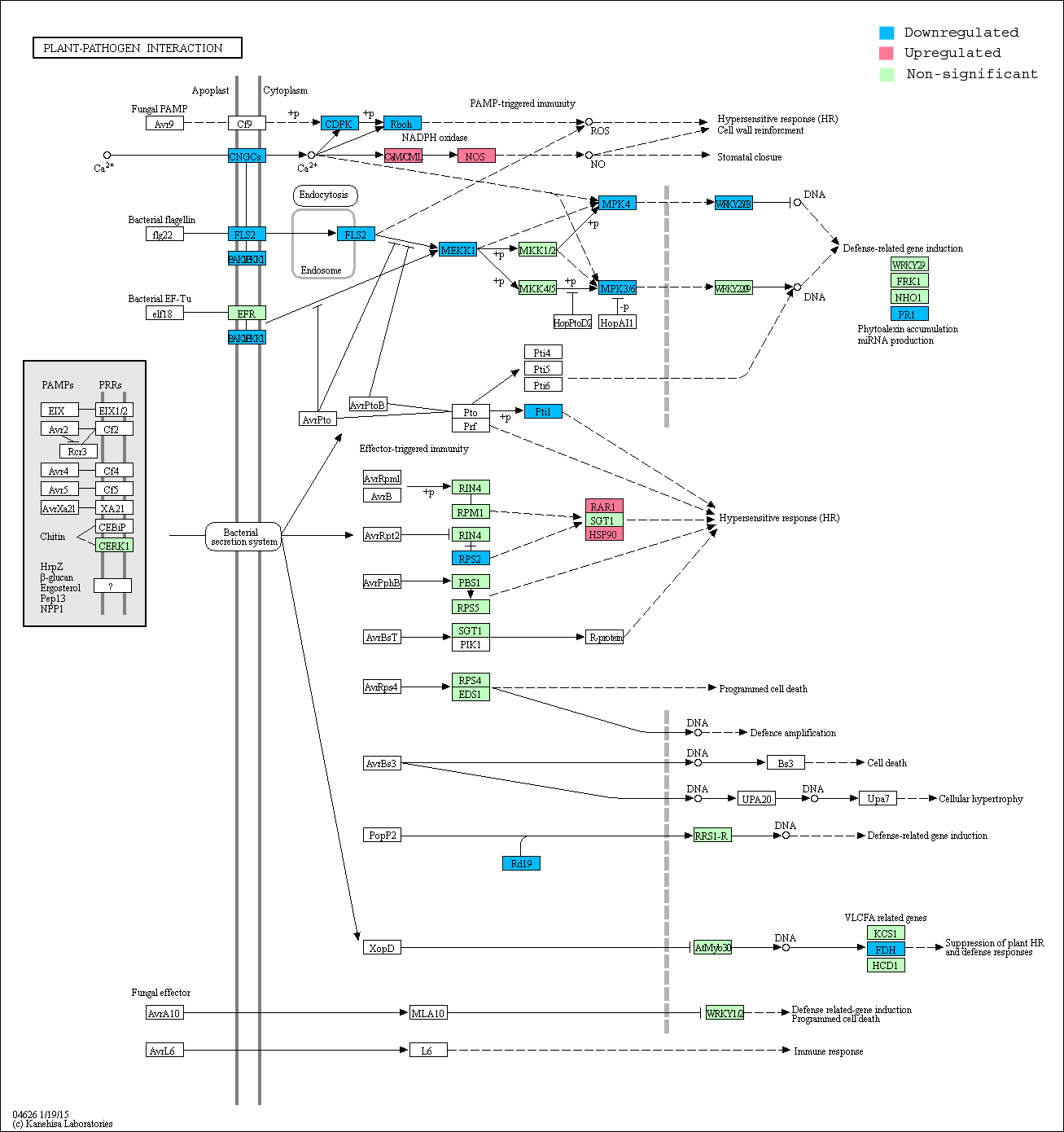


**Figure S14.** Plant-pathogen interaction pathway analysis performed using online tool KOBAS. Color key on the top right indicates the downregulation (blue), upregulation (pink) and non-significant regulation (green) tn Mac7 RNA-Seq data

**Supplementary Tables**

**Table S1.** RNA-Seq samples under each treatment with number of raw and trimmed reads, percent mapping and GC, and average Phred score.

| **No** | **Sample** | **Condition** | **No. of reads (raw)** | **No. of reads (trimmed)** | **% Mapping** | **%GC** | **Avg. Phred** |
| --- | --- | --- | --- | --- | --- | --- | --- |
| 1 | RS-1 | CLCuV-infested | 30633575 | 29170717 | 71.71 | 43 | 37 |
| 2 | RS-2 | CLCuV-infested | 27278557 | 26253194 | 71.69 | 44 | 37 |
| 3 | RS-3 | CLCuV-infested | 31927643 | 30792360 | 73.12 | 44 | 37 |
| 4 | RS-4 | CLCuV-infested | 26120674 | 25747058 | 75.39 | 44 | 37 |
| 5 | RS-5 | CLCuV-free | 29147002 | 27445162 | 72.49 | 44 | 37 |
| 6 | RS-6 | CLCuV-free | 28748496 | 28649743 | 64.87 | 42 | 37 |
| 7 | RS-7 | CLCuV-free | 25707191 | 22762310 | 73.01 | 44 | 37 |
| 8 | RS-8 | CLCuV-free | 29046579 | 27897815 | 73.04 | 44 | 37 |

**Table S2.** FPKMs of housekeeping genes in RNA-Seq data with respective gene IDs, p-values and q-values.

| **No.** | **Gene_function** | **gene_id** | **FPKM_Con** | **FPKM_Inf** | **log2(fold_change)** | **p_value** | **q_value** | **significant** |
| --- | --- | --- | --- | --- | --- | --- | --- | --- |
| 1 | AGP | CotAD_04723 | 1.19055 | 0.336809 | -1.82162 | 0.04835 | 0.106246 | no |
| 2 | 18S rRNA | CotAD_06141 | 29.2726 | 30.413 | 0.0551355 | 0.8153 | 0.874773 | no |
| 3 | Betatubulin | CotAD_18492 | 91.1559 | 90.5668 | -0.00935462 | 0.96605 | 0.977367 | no |
| 4 | ubiquitin | CotAD_22588 | 36.0391 | 29.3036 | -0.298485 | 0.19615 | 0.310908 | no |
| 5 | carboxyl-terminal hydrolase 6 | CotAD_42259 | 30.3054 | 24.733 | -0.293142 | 0.1887 | 0.301791 | no |
| 6 | stearoyl-[acyl-carrier-protein] 9-desaturase | CotAD_55670 | 93.5876 | 90.7587 | -0.044283 | 0.8423 | 0.893786 | no |
| 7 | Actin | CotAD_75356 | 21.5375 | 17.3562 | -0.3114 | 0.1954 | 0.309878 | no |
| 8 | CYP1 | CotAD_47020 | 544.492 | 546.828 | 0.00617551 | 0.9813 | 0.986886 | no |

**Table S3.** Differentially expressed genes in transcriptomic data of CLCUD infested versus disease free Mac7, with respective gene IDs, FPKMs, log2FC, p-values and q-values.

(Table S3 is attached as online supplementary information)

**Table S4.** List of primers used in this study for qPCR validation of RNA-Seq data

| **No.** | **Primer name** | **Primer sequence** | **Gene ID** |
| --- | --- | --- | --- |
| 1 | RS97 | GCCTGGCCATACTGATCCTA | CotAD_63233 |
| 2 | RS98 | ACAGCTCGATGGAGCAGACT |  |
| 3 | RS99 | CAAGGATGTTCCAGGGCTTA | CotAD_69847 |
| 4 | RS100 | GTGGCTCGTAGCTTTTCAGG |  |
| 5 | RS101 | CCCATTCCCTTCTTCACTCA | CotAD_12715 |
| 6 | RS102 | GATTTGAAGCACCCTGTCGT |  |
| 7 | RS103 | ATTTCGATGCCAGTGGTAGG | CotAD_27998 |
| 8 | RS104 | CACCATGGGGATGTTAAACC |  |
| 9 | RS105 | GTGCCTGGTTTCAACCTCAT | CotAD_42837 |
| 10 | RS106 | CCAGCCCTGTTCTTAAGTCG |  |
| 11 | RS107 | TATCAGCAATGCCTCTGACG | CotAD_57854 |
| 12 | RS108 | TCCTTGGTACCAGACCTTGC |  |
| 13 | RS111 | GTCGAGCGAAAATGGAACAT | CotAD_02055 |
| 14 | RS112 | CATCATGGCAAACGTGTAGG |  |
| 15 | RS113 | TCTCACCTTCAACCCCAAAG | CotAD_10536 |
| 16 | RS114 | ACCATACCCGAATCCCTTTC |  |
| 17 | RS117 | TCCTCTGAGATTCGCAGCTT | CotAD_73181 |
| 18 | RS118 | ATCCCAGCTGAACGATGAGA |  |
| 19 | RS121 | CGGAGACTCGGTTGAAGAAG | CotAD_71021 |
| 20 | RS122 | GTGCCACCATTCTTTCCAGT |  |
| 21 | RS123 | GGAGTAGCGCTGACTTTTCG | CotAD_44575 |
| 22 | RS124 | TGCACCTCACCTTAGCACAC |  |
| 23 | RS125 | ATTGTTCCAGGGTGCCATAG | CotAD_52325 |
| 24 | RS126 | CCTCTGCATTCTTTCCAAGG |  |
| 25 | RS127 | TTATCAAGGGCTGGGATGAG | CotAD_26548 |
| 26 | RS128 | CCATTTCTCCCCTTCAACAA |  |
| 27 | RS129 | GCCTGAGACGGCTGAAATAG | CotAD_48246 |
| 28 | RS130 | ACTCGATCCGAATCAGGTTG |  |
| 29 | RS131 | CATACGGCAACTTGCTCAGA | CotAD_06825 |
| 30 | RS132 | TTGCCAATCCTTCTTGTTCC |  |
| 31 | RS133 | CCACGGTTCTTCATGTTGTG | CotAD_60956 |
| 32 | RS134 | TACCAACACCATAGCCACGA |  |
| 33 | RS135 | TAACAACCAGGGGCTTCAAC | CotAD_13867 |
| 34 | RS136 | GTGACGGACACTTGCTGAGA |  |
| 35 | RS139 | TCAAGGTCCAAGTGGAGGAC | CotAD_00296 |
| 36 | RS140 | CCGTCTTGACAGATTGCAGA |  |
| 37 | RS143 | TGGGCTTTACCGATTTGTTC | CotAD_09671 |
| 38 | RS144 | GGACGATCCTAGGGTGGATT |  |
| 39 | RS145 | ATGGGTTCATTGAGCAAAGC | CotAD_52383 |
| 40 | RS146 | GGAATTGGGTCAGGGAATTT |  |
| 41 | RS147 | TCCATGGAGATGGGAGAGAC | CotAD_25281 |
| 42 | RS148 | TCCTCTTCAGCAACCCATCT |  |
| 43 | RS149 | TGGATTTCCAAAGCCAAAAA | CotAD_16873 |
| 44 | RS150 | ACCAAATCGCCACTTGTCTC |  |
| 45 | RS151 | TTGGATGAGGAAACCAGAGG | CotAD_52090 |
| 46 | RS152 | AAAGGGTGCCCTCTTAGGAA |  |
| 47 | RS153 | AGCCCTTGCTTCATGTGTTT | CotAD_28280 |
| 48 | RS154 | CGAGGCCAGTAGCATAGAGG |  |

**Table S5.** Network analyses for resist mock

(Table S5 is attached as online supplementary information)

**Table S6.** Information centrality analysis

(Table S6 is attached as online supplementary information)

**Table S7.** GO terms associated with highly connected 52 hubs identified in WGCNA, with respective p values.

(Table S7 is attached as online supplementary information)

**Table S8.** Differentially expressed genes in transcriptomic data of CLCUD-susceptible cotton line (karishma) versus CLCuD-resistant Mac-7, with respective gene IDs and log2FC FPKMs.

**Table S9. Genes selected for virus-induced gene silencing (VIGS) experiment and their respective primers**

| **Gene_ID** | **Description** | **Product Size** | **Primers** |
| --- | --- | --- | --- |
| CotAD_71021 | Serine/threonine-protein kinase | 220 | CGGAGACTCGGTTGAAGAAG |
|  |  |  | GTGCCACCATTCTTTCCAGT |
| CotAD_48246 | E3 ubiquitin-protein ligase | 177 | GCCTGAGACGGCTGAAATAG |
|  |  |  | ACTCGATCCGAATCAGGTTG |
|  |  |  | CCGTCTTGACAGATTGCAGA |
| CotAD_52090 | Heat shock cognate protein 80 | 160 | TTGGATGAGGAAACCAGAGG |
|  |  |  | AAAGGGTGCCCTCTTAGGAA |

**Table S10. List of differentially expressed genes identified in RNA-Seq and confirmed with qPCR.**

Log2 fold change (FC) of Fragments Per Kilobase of Transcript per Million mapped reads (FPKM) in the RNA-Seq experiment is provided in last column.

| **No.** | **Gene ID** | **Chromosome location** | **Putative function** | **FPKM_Con** | **FPKM_Inf** | **log2FC** | **p_value** | **q_value** |
| --- | --- | --- | --- | --- | --- | --- | --- | --- |
| 1 | 00296 | Dt_chr9:66061420..66061890 - | Heat shock protein - Class II | 11.1776 | 672.041 | 5.90987 | 5.00E-05 | 0.000327 |
| 2 | 02055 | Dt_chr3:16036977..16038985 + | Protein NRT1/ PTR family | 8.44655 | 0.114834 | -6.20074 | 0.00335 | 0.012249 |
| 3 | 06825 | At_chr13:24968896..24970310 - | Heat shock factor protein HSF30 isoform X1 | 4.17763 | 447.356 | 6.74259 | 5.00E-05 | 0.000327 |
| 4 | 09671 | Dt_chr9:58149876..58151220 - | Inorganic pyrophosphatase 1 | 52.9814 | 0.98952 | -5.74261 | 5.00E-05 | 0.000327 |
| 5 | 10536 | Dt_chr1:40959566..40960489 + | Ethylene-responsive transcription factor | 20.698 | 0.200012 | -6.69326 | 0.00345 | 0.012559 |
| 6 | 12715 | Dt_chr8:39643677..39644150 - | Heat shock protein - Class I | 4.05213 | 963.783 | 7.89389 | 5.00E-05 | 0.000327 |
| 7 | 13867 | Dt_chr12:2829302..2832473 - | Ankyrin 3 | 13.6898 | 0.248865 | -5.78159 | 5.00E-05 | 0.000327 |
| 8 | 16873 | At_chr2:7666817..7667350 - | Homeobox-leucine zipper protein | 124.261 | 2.049 | -5.92231 | 5.00E-05 | 0.000327 |
| 9 | 25281 | Dt_chr7:22345516..22349212 - | 3-epi-6-deoxocathasterone 23-monooxygenase | 7.9025 | 0.127887 | -5.94937 | 0.00355 | 0.01287 |
| 10 | 26548 | Dt_chr6:3140078..3143328 + | Peptidyl-prolyl cis-trans isomerase | 6.11606 | 351.985 | 5.84677 | 5.00E-05 | 0.000327 |
| 11 | 27998 | Dt_chr8:52457718..52458446 + | Osmotin-like pathogenesis-related protein | 631.453 | 3.18588 | -7.63084 | 5.00E-05 | 0.000327 |
| 12 | 28280 | Dt_chr2:49515745..49516482 - | Probable F-box protein | 0.767322 | 43.732 | 5.83271 | 5.00E-05 | 0.000327 |
| 13 | 42837 | Dt_chr8:56482356..56484173 + | Carotenoid cleavage dioxygenase 4 | 19.3338 | 0.130739 | -7.20829 | 5.00E-05 | 0.000327 |
| 14 | 44575 | At_chr6:46895744..46896091 - | Pathogenesis-related protein | 161.028 | 0.737577 | -7.77031 | 0.0163 | 0.044539 |
| 15 | 48246 | Dt_chr6:1960039..1960563 + | E3 ubiquitin-protein ligase | 1.0931 | 108.926 | 6.63877 | 5.00E-05 | 0.000327 |
| 16 | 52090 | Dt_chr13:13115211..13117847 + | Heat shock cognate protein 80 | 11.5308 | 577.653 | 5.64664 | 5.00E-05 | 0.000327 |
| 17 | 52325 | At_chr6:21237843..21239411 + | Transcription factor BEE 1-like | 13.8159 | 0.216235 | -5.99759 | 0.0139 | 0.039133 |
| 18 | 52383 | Dt_chr5:20258487..20260348 + | Serine/threonine-protein kinase | 1.08625 | 122.658 | 6.81914 | 5.00E-05 | 0.000327 |
| 19 | 57854 | Dt_chr8:55666269..55669224 + | Heat shock protein 83 | 0.862943 | 254.828 | 8.20604 | 5.00E-05 | 0.000327 |
| 20 | 60956 | At_chr13:77673979..77676458 + | Pheophytinase | 10.8023 | 0.115997 | -6.54111 | 0.0155 | 0.042727 |
| 21 | 63233 | Dt_chr4:14434861..14436182 - | Protein DMR6-LIKE OXYGENASE 2 | 0.927378 | 22.5086 | 4.60118 | 5.00E-05 | 0.000327 |
| 22 | 69847 | At_chr8:67853466..67856507 + | Chalcone synthase | 25.4617 | 0.438219 | -5.86053 | 5.00E-05 | 0.000327 |
| 23 | 71021 | At_chr5:46254768..46256610 - | Serine/threonine-protein kinase | 0.765681 | 74.1308 | 6.59719 | 0.00025 | 0.001375 |
| 24 | 73181 | At_chr9:45390209..45391409 + | Calcyclin-binding protein | 0.395557 | 9.32651 | 4.55938 | 0.00235 | 0.009102 |

**References**

Carvalho, C.M., Fontenelle, M.R., Florentino, L.H., Santos, A.A., Zerbini, F.M. and Fontes, E.P. (2008a) A novel nucleocytoplasmic traffic GTPase identified as a functional target of the bipartite geminivirus nuclear shuttle protein. *Plant J* **55**, 869-880.

Carvalho, C.M., Machado, J.P.B., Zerbini, F.M. and Fontes, E.P.B. (2008b) NSP-Interacting GTPase: A cytosolic protein as cofactor for nuclear shuttle proteins. *Plant Signal Behav* **3**, 752-754.

Carvalho, C.M., Santos, A.A., Pires, S.R., Rocha, C.S., Saraiva, D.I., Machado, J.P., Mattos, E.C., Fietto, L.G. and Fontes, E.P. (2008c) Regulated nuclear trafficking of rpL10A mediated by NIK1 represents a defense strategy of plant cells against virus. *PLoS Pathog* **4**, e1000247.

Dogra, S.C., Eini, O., Rezaian, M.A. and Randles, J.W. (2009) A novel shaggy-like kinase interacts with the *Tomato leaf curl virus* pathogenicity determinant C4 protein. *Plant Mol Biol* **71**, 25-38.

Fontes, E.P., Santos, A.A., Luz, D.F., Waclawovsky, A.J. and Chory, J. (2004) The geminivirus nuclear shuttle protein is a virulence factor that suppresses transmembrane receptor kinase activity. *Genes Dev* **18**, 2545-2556.

Lai, J., Chen, H., Teng, K., Zhao, Q., Zhang, Z., Li, Y., Liang, L., Xia, R., Wu, Y., Guo, H. and Xie, Q. (2009) RKP, a RING finger E3 ligase induced by BSCTV C4 protein, affects geminivirus infection by regulation of the plant cell cycle. *Plant J* **57**, 905-917.

Lozano-Duran, R., Rosas-Diaz, T., Luna, A.P. and Bejarano, E.R. (2011) Identification of host genes involved in geminivirus infection using a reverse genetics approach. *PLoS One* **6**, e22383.

Piroux, N., Saunders, K., Page, A. and Stanley, J. (2007) Geminivirus pathogenicity protein C4 interacts with *Arabidopsis thaliana* shaggy-related protein kinase AtSKeta, a component of the brassinosteroid signalling pathway. *Virology* **362**, 428-440.

Rocha, C.S., Santos, A.A., Machado, J.P. and Fontes, E.P. (2008) The ribosomal protein L10/QM-like protein is a component of the NIK-mediated antiviral signaling. *Virology* **380**, 165-169.

Sanderfoot, A.A. and Lazarowitz, S.G. (1995) Cooperation in viral movement: the geminivirus BL1 movement protein interacts with BR1 and redirects it from the nucleus to the periphery. *Plant Cell* **7**, 1185-1194.

Selth, L.A., Dogra, S.C., Rasheed, M.S., Healy, H., Randles, J.W. and Rezaian, M.A. (2005) A NAC domain protein interacts with *Tomato leaf curl virus* replication accessory protein and enhances viral replication. *Plant Cell* **17**, 311-325.
